# Glycoprotein NMB mediates bidirectional GSC-TAM interactions to promote tumor progression

**DOI:** 10.1172/jci.insight.187684

**Published:** 2025-07-08

**Authors:** Yang Liu, Lizhi Pang, Fatima Khan, Junyan Wu, Fei Zhou, Craig Horbinski, Shideng Bao, Jennifer S. Yu, Justin D. Lathia, Peiwen Chen

**Affiliations:** 1 Department of Cancer Biology, Lerner Research Institute, Cleveland Clinic, Cleveland, Ohio, USA.; 2 Department of Neurological Surgery, Feinberg School of Medicine, Northwestern University, Chicago, Illinois, USA.; 3 Case Comprehensive Cancer Center, Cleveland, Ohio, USA.; 4 Department of Cardiovascular and Metabolic Sciences, Lerner Research Institute, Cleveland Clinic, Cleveland, Ohio, USA.

**Keywords:** Immunology, Oncology, Brain cancer

## Abstract

Glioblastoma (GBM) is a lethal brain tumor containing a subpopulation of GBM stem cells (GSCs) that interaction with surrounding cells, including infiltrating tumor-associated macrophages and microglia (TAMs). While GSCs and TAMs are in close proximity and likely interact to coordinate tumor growth, a limited number of mechanisms have been identified that support their communication. Here, we identified glycoprotein NMB (GPNMB) as a key factor mediating a unique bidirectional interaction between GSCs and TAMs in GBM. Specifically, GSCs educated macrophages and microglia to preferentially express GPNMB in the GBM tumor microenvironment. As a result, TAM-secreted GPNMB interacted with its receptor CD44 on GSCs to promote their glycolytic and self-renewal abilities via activating the PYK2/RSK2 signaling axis. Disrupting GPNMB-mediated GSC-TAM interplay suppressed tumor progression and self-renewal in GBM mouse models. Our study found a protumor function of GPNMB-mediated GSC-TAM bidirectional communication and supports GPNMB as a promising therapeutic target for GBM.

## Abstract 2

GPNMB-mediated GSC-TAM crosstalk promotes GBM progression and stemness via activating the CD44-PYK2-RSK2 signaling axis.

## Introduction

Glioblastoma (GBM) is an aggressive and lethal brain tumor, lacking effective curative treatments. Despite numerous clinical trials that have been developed over the last two decades, the progress for GBM management is still limited (1, 2). The current standard of care for GBM includes maximal surgical resection of primary tumors followed by radiation and chemotherapy with temozolomide (3, 4). Even with these aggressive treatments, the prognosis of patients with GBM remains dismal, with median overall survival rate and 5-year survival rate of only about 15–20 months and 10%, respectively, after initial diagnosis (1, 2). GBM stem cells (GSCs) are a subpopulation of GBM cells in the tumor microenvironment (TME) that are characterized by stem cell–like capabilities (5). The presence of GSCs underlies GBM intratumoral heterogeneity and treatment resistance (5). Despite advances in our understanding of the functional properties of GSCs, the mechanisms underlying their pathogenesis and maintenance, including the relationships between GSCs and other cell populations within the GBM TME, are yet to be fully elucidated.

Recent studies on the GBM TME have revealed that the low immunogenicity also generates tumor heterogeneity, promotes tumor progression, and results in treatment resistance (6, 7). The GBM TME harbors distinct immune cell populations, such as tumor-associated macrophages and microglia (TAMs), myeloid-derived suppressor cells, DCs, neutrophils, and T cells (6). Among them, TAMs are the largest population of immune cells, representing as much as 50% of total cells in the whole GBM tumor mass and encompassing bone marrow–derived macrophages (BMDMs) and yolk sac–derived microglia (8, 9). The recent research focusing on context-dependent tumor-TAM bidirectional interactions reveals that the signaling from GSCs/GBM cells not only influences the malignancy of cancer cells intrinsically, but also affects TAM biology (6, 8, 10, 11). Indeed, the crosstalk between GSCs and TAMs is an important mechanism within the GBM TME that promotes immune escape of GBM tumors (6, 8). For example, *Nf1*-deficient GSCs isolated from tumors of the *Nf1* genetically engineered mouse model can secrete chemokines, such as CX3CL1 and CCL5, to recruit immunosuppressive microglia into the TME (12). Furthermore, our recent studies demonstrated that *PTEN* deletion/mutation and metabolic alteration in GSC/GBM cells can activate the SRC/AKT-YAP1 and YAP1/STAT3 pathways to upregulate the expression of chemokines (e.g., lysyl oxidase, CCL2, and CCL7), triggering macrophage migration and promoting tumor progression (13, 14). In addition, amplification and overexpression of circadian locomotor output cycles kaput (CLOCK) in GSCs can transcriptionally upregulate the expression and secretion of olfactomedin like 3 (OLFML3) and legumain (LGMN) to stimulate microglia infiltration and immunosuppressive polarization, generating an environment promoting tumor growth (15, 16). Kunitz-type protease inhibitor TFPI2 is another example that is amplified in about 4% of patients with GBM and can be secreted by GSCs to trigger microglia infiltration and immunosuppressive polarization, thus inhibiting T cell antitumor immunity and promoting tumor progression (17). Together, these findings emphasize the importance of several GSC-derived factors that mediate the interplay between tumor cells and TAMs to promote GBM progression. However, these interactions are likely bidirectional, and the role of TAM-derived factors in this process is largely unknown. In the current study, we have conducted comprehensive analyses and studies aimed at identifying TAM-specific factors that can respond to tumor stimuli and then act on GSCs, forming a protumorigenic TAM/GSC signaling network. In this investigation, glycoprotein NMB (GPNMB) emerged as the top hit.

GPNMB, a transmembrane glycoprotein, has been identified for its elevated expression in a melanoma cell line with limited metastatic tendencies (18). Due to its involvement in promoting osteoblast differentiation and bone mineral deposition, it is alternatively referred to as osteoactivin (19, 20). The membrane-bound part of GPNMB can be cleaved by metalloproteinases, generating a soluble isoform that binds to its receptors on different types of cells to facilitate cellular communications (21, 22). GPNMB is expressed in tumors across cancer types, such as breast cancer, melanoma, hepatocellular carcinoma, prostate cancer, osteosarcoma, and lung cancer, where it plays an important role in regulating cancer cell migration, invasion, metastasis, and treatment resistance (23–28). Recent evidence also shows that GPNMB is implicated in tumor immune evasion by directly inhibiting T cell activation in melanoma (29). In GBM, GPNMB has been shown to be highly expressed in TAMs, where it can promote tumor growth and impede T cell activation (30–32). However, the potential roles of TAM-derived GPNMB in GSC stemness maintenance and the GSC-TAM bidirectional interaction have not been studied.

Here, we showed that GSCs educate macrophages and microglia to preferentially express GPNMB in the GBM TME. TAM-derived GPNMB promoted GSC glycolysis and self-renewal via the activation of the CD44-proline-rich tyrosine kinase 2 (PYK2)/p90 ribosomal S6 kinase 2 (RSK2) signaling axis. Blockade of the GSC-TAM bidirectional interaction via depleting TAM GPNMB suppressed GBM growth and reduced GSC stemness. Altogether, our study deciphers the mechanism of GPNMB in mediating GSC-TAM crosstalk and provides a potential strategy to target this unique bidirectional in GBM.

## Results

### GSCs educate macrophages and microglia to upregulate GPNMB expression.

**Figure 1:**
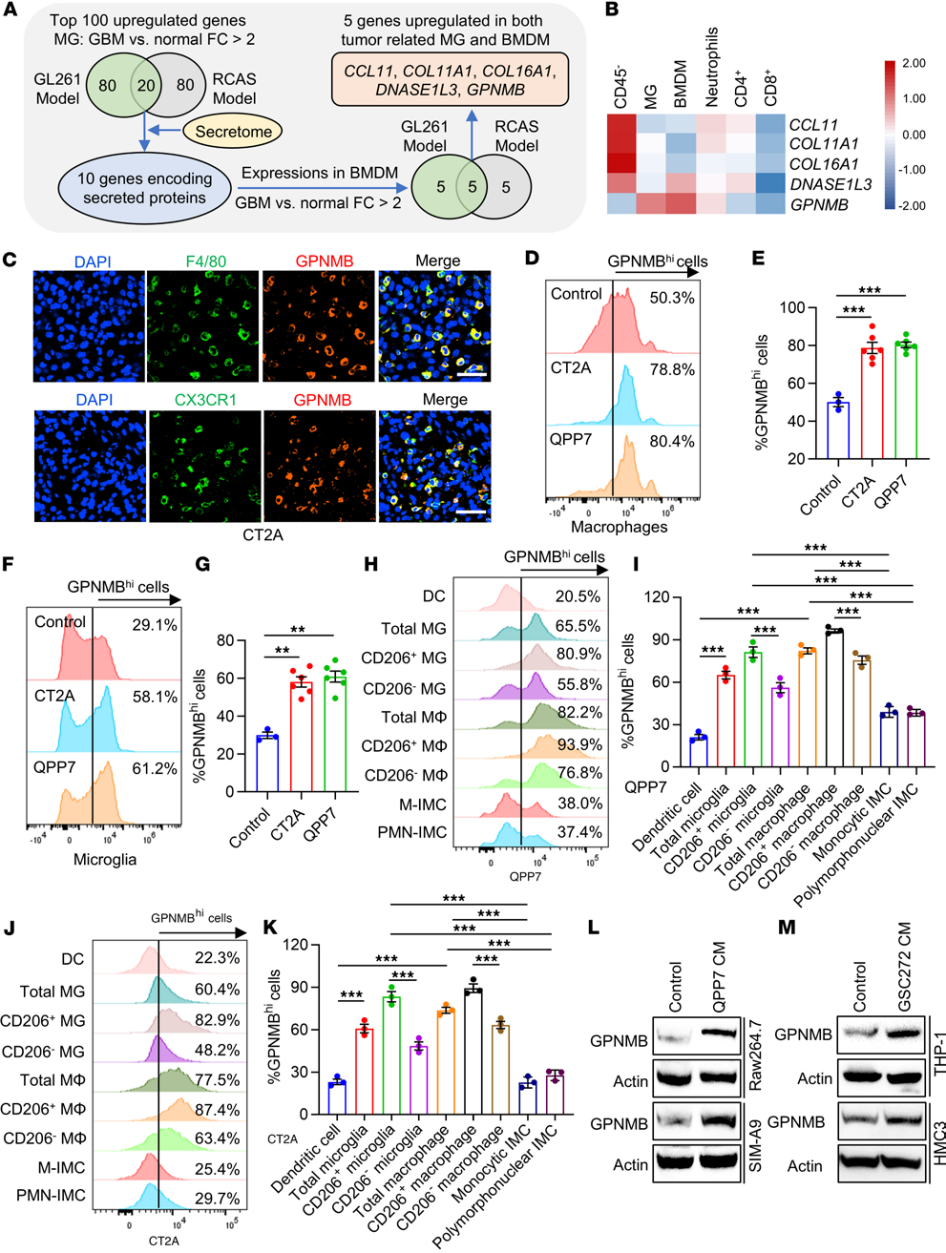
GSCs upregulate GPNMB expression in TAMs. (**A**) Strategy for identification of the overlapped genes that were upregulated in both GBM-associated microglia and bone marrow–derived macrophage (BMDMs) compared with normal monocytes. The analysis was based on RNA-Seq data (GSE86573) from GBM-associated microglia/BMDMs isolated from the GL261 tumor model and RCAS tumor model and normal microglia and monocytes. (**B**) The expression pattern of genes in CD45^–^ GBM cells and tumor-associated immune cells (e.g., BMDMs, microglia, neutrophils, CD4^+^ T cells, and CD8^+^ T cells) isolated from human GBM tumors based on the Brain TIME dataset (34). (**C**) Coimmunofluorescence staining for GPNMB (red) and F4/80 (macrophage marker, green) or CX3CR1 (microglia marker, green) in CT2A tumors implanted in C57BL/6 mice. Scale bar: 50 μm. (**D**–**G**) Flow cytometry analysis of GPNMB expression in CD11b^+^CD45^hi^CD68^+^ macrophages (**D** and **E**) isolated from bone marrow and CD11b^+^CD45^lo^CX3CR1^+^ microglia (**F** and **G**) isolated from brain tissues of tumor-free C57BL/6 mice and CT2A and QPP7 tumor-bearing C57BL/6 mice. *n* = 3–6 independent samples. One-way ANOVA test. (**H**–**K**) Flow cytometry analysis of GPNMB expression in CD11b^+^CD45^hi^CD11c^+^ DCs, CD11b^+^CD45^lo^CX3CR1^+^ microglia (Total MG), CD11b^+^CD45^lo^CX3CR1^+^CD206^+^ microglia (CD206^+^ MG), CD11b^+^CD45^lo^CX3CR1^+^CD206^–^ microglia (CD206^–^ MG), CD11b^+^CD45^hi^CD68^+^ macrophages (Total MΦ), CD11b^+^CD45^hi^CD68^+^CD206^+^ macrophages (CD206^+^ MΦ), CD11b^+^CD45^hi^CD68^+^CD206^–^ macrophages (CD206^–^ MΦ), CD11b^+^CD68^+^Ly6G^lo^Ly6C^hi^ monocytic immature myeloid cells (M-IMCs), and CD11b^+^CD68^+^Ly6G^hi^Ly6C^lo^ polymorphonuclear immature myeloid cells (PMN-IMCs) isolated from QPP7 tumors (**H** and **I**) and CT2A tumors (**J** and **K**) implanted in C57BL/6 mice. *n* = 3 independent samples. One-way ANOVA test. (**L**) Immunoblots for GPNMB in lysates of Raw264.7 macrophages and SIM-A9 microglia treated with the conditioned media (CM) of QPP7 GSCs for 24 hours. (**M**) Immunoblots for GPNMB in lysates of THP-1 macrophage and HMC3 microglia treated with the CM of GSC272 cells for 24 hours. **P < 0.01, ***P < 0.001.

To identify potential factors that are highly expressed in TAMs and might mediate the GBM-TAM bidirectional interaction, we analyzed RNA-Seq data of GBM-associated BMDMs and microglia isolated from tumors of GL261 and RCAS (a genetically engineered mouse model with *Pdgfb* induction and *Trp53* knockdown) models (33). We found that 5 genes (*CCL11*, *COL11A1*, *COL16A1*, *DNASE1L3*, and *GPNMB*) encoding secreted proteins were upregulated in both GBM-associated microglia and BMDMs compared with normal microglia and monocytes, respectively (Figure 1A). Next, we used the Brain Tumor Immune Micro Environment (TIME) dataset to analyze the expression levels of these 5 genes in CD45^–^ GBM cells and tumor-associated immune cells (e.g., BMDMs, microglia, neutrophils, CD4^+^ T cells, and CD8^+^ T cells) isolated from human GBM tumors (34). The results showed that *GPNMB*, but not the other 4 genes, was highly expressed in both microglia and BMDMs (Figure 1B). To further confirm this expression pattern, we performed immunofluorescence staining in tumors from C57BL/6J mice implanted with CT2A cells, an AKT-activated GBM cell line possessing GSC-like phenotype (15, 17). The results showed that GPNMB was predominantly expressed in F4/80^+^ macrophages and CX3CR1^+^ microglia, while its expression was comparatively lower in immature myeloid cells (IMCs), including Ly6C^+^ monocytic and Ly6G^+^ polymorphonuclear IMCs (Figure 1C and Supplemental Figure 1A; supplemental material available online with this article; https://doi.org/10.1172/jci.insight.187684DS1). Next, we performed flow cytometry for distinct types of immune cells in tumors implanted with QPP7 GSCs, a GSC line isolated from spontaneous tumors of GBM mouse model with null alleles for *Pten*, *Qki*, and *Trp53* (35), and CT2A cells. Compared with control primary mouse BMDMs and microglia, GSC tumor-associated CD11b^+^CD45^hi^CD68^+^ macrophages and CD11b^+^CD45^lo^CX3CR1^+^ microglia showed a higher GPNMB expression in both models (Figure 1, D–G). Within the tumor-associated myeloid cells, CD11b^+^CD45^hi^CD68^+^ macrophages and CD11b^+^CD45^lo^CX3CR1^+^ microglia exhibited higher GPNMB expression compared with that of CD11b^+^CD45^hi^CD11c^+^ DCs, CD11b^+^CD68^+^Ly6G^lo^Ly6C^hi^ monocytic IMCs, and CD11b^+^CD68^+^Ly6G^hi^Ly6C^lo^ polymorphonuclear IMCs (Figure 1, H–K). Notably, CD11b^+^CD45^hi^CD68^+^CD206^+^ protumor macrophages and CD11b^+^CD45^lo^CX3CR1^+^CD206^+^ protumor microglia showed the highest GPNMB expression among myeloid cell populations in QPP7 and CT2A tumors (Figure 1, H–K). To confirm the potential role of GSCs in upregulating GPNMB expression in TAMs, we performed immunoblotting experiments in macrophages and microglia treated with or without conditioned media (CM) of GSCs. The results showed that CM from mouse QPP7 GSCs and CT2A cells upregulated the expression of GPNMB in mouse Raw264.7 macrophages and SIM-A9 microglia (Figure 1L and Supplemental Figure 1B). Similar effects were observed in human THP-1 macrophages and HMC3 microglia when they were treated with the CM from GSC272 and GSC2 cells derived from patients with GBM (Figure 1M and Supplemental Figure 1C). Together, these findings suggest that GPNMB is highly expressed in GBM TAMs upon the education by GSCs.

### TAM-derived GPNMB promotes GSC glycolysis.

Given *GPNMB* is a gene encoding secreted protein, we further examined whether GSCs affect GPNMB secretion from macrophages and microglia. An ELISA assay demonstrated that GPNMB levels in the CM of THP-1 macrophages and HMC3 microglia were upregulated upon the treatment with GSC272 cell CM (Supplemental Figure 1, D and E). Moreover, we compared GPNMB levels in patient plasma and found that GPNMB was higher in patients with GBM than in healthy individuals acting as controls (Supplemental Figure 1F). This expression pattern was consistent with the level of macrophages and microglia signatures in samples from healthy individuals acting as controls and patients with GBM (36) (Supplemental Figure 1, G and H). These findings suggest that GPNMB can be secreted from GSC-educated macrophages and microglia, which, in turn, might play an important role in mediating the communication between TAMs and GSCs.

**Figure 2:**
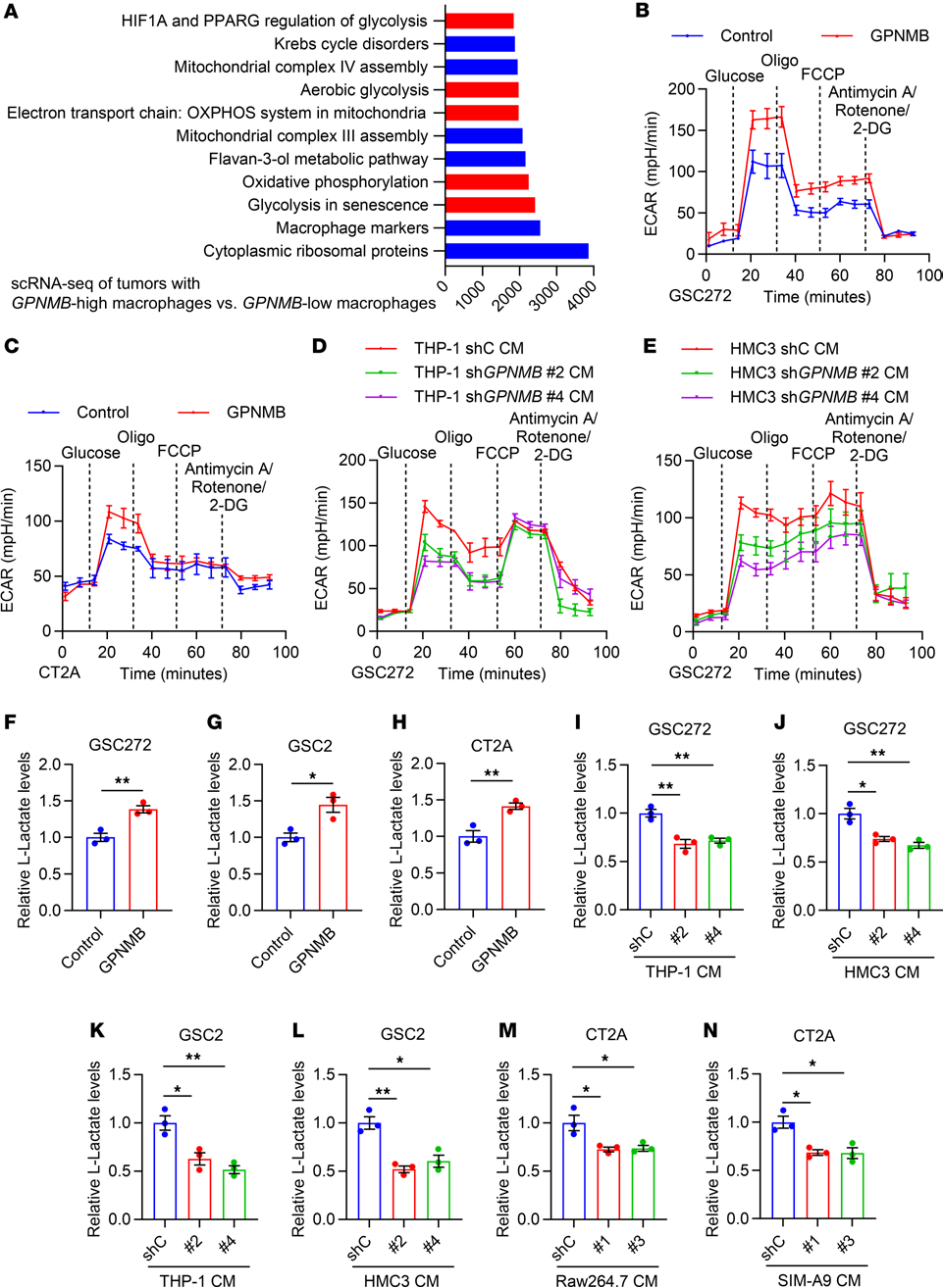
GPNMB promotes the glycolysis of GSCs. (**A**) GSEA analysis of single-cell RNA-Seq (scRNA-Seq) data from human GBM tumors (EGAS00001004871) shows top enriched WikiPathways signatures in tumors with TAMs expressing high *GPNMB* compared to low *GPNMB*. (**B** and **C**) Extracellular acidification rate (ECAR) of GSC272 cells (**B**) and CT2A cells (**C**) treated with GPNMB recombinant protein (100 ng/mL) for 24 hours. ECAR was obtained from the Seahorse experiments, and glucose was added at the indicated time point. *n* = 3 independent samples. (**D** and **E**) ECAR of GSC272 cells treated with the conditioned media (CM) of THP-1 macrophages (**D**) and HMC3 microglia (**E**) expressing shRNA control (shC) or *GPNMB* shRNA (sh*GPNMB*) for 24 hours. ECAR was obtained from the Seahorse experiments, and glucose was added at the indicated time point. *n* = 6 independent samples. (**F**–**H**) Quantification of relative L-lactate levels in GSC272 (**F**), GSC2 (**G**), and CT2A cells (**H**) treated with GPNMB recombinant protein (100 ng/mL) for 24 hours. *n* = 3 independent samples. Student’s *t* test. (**I** and **J**) Quantification of relative L-lactate levels in GSC272 cells treated with the CM of THP-1 macrophages (**I**) and HMC3 microglia (**J**) expressing shC or sh*GPNMB* for 24 hours. *n* = 3 independent samples. One-way ANOVA test. (**K** and **L**) Quantification of relative L-lactate levels in GSC2 cells treated with the CM of THP-1 macrophages (**K**) and HMC3 microglia (**L**) expressing shC or sh*GPNMB* for 24 hours. *n* = 3 independent samples. One-way ANOVA test. (**M** and **N**) Quantification of relative L-lactate levels in CT2A cells treated with the CM of Raw264.7 macrophages (**M**) and SIM-A9 microglia (**N**) expressing shC or sh*Gpnmb* for 24 hours. *n* = 3 independent samples. One-way ANOVA test. *P < 0.05, **P < 0.01.

To explore the function of TAM-derived GPNMB on tumor biology, we performed gene set enrichment analysis (GSEA) on WikiPathways using the single-cell RNA-Seq (scRNA-Seq) data of GBM tumors (37). Compared with tumors with *GPNMB*-low macrophages, tumors with *GPNMB*-high macrophages displayed an increased enrichment of several metabolic pathways, including glycolysis (Figure 2A and Supplemental Figure 2A), supporting a potential role of TAM-derived GPNMB in affecting GSC glycolysis. Indeed, we observed an enhanced glycolytic activity, as shown by increased the extracellular acidification rate (ECAR) in human GSC272 cells and mouse CT2A cells treated with GPNMB recombinant protein (Figure 2, B and C). In contrast, CM from GPNMB-depleted THP-1 macrophages and HMC3 microglia significantly impaired the ECAR in GSC272 cells when compared with the CM from control macrophages and microglia (Figure 2, D and E, and Supplemental Figure 2, B and C). Next, we examined lactate levels in human and mouse GSCs and found that lactate was increased upon the treatment with GPNMB recombinant protein (Figure 2, F–H) but was decreased by the treatment with CM from GPNMB-depleted macrophages (e.g., human THP-1 and mouse Raw264.7) and microglia (e.g., human HMC3 and mouse SIM-A9) compared with the CM from shRNA controls (shC) (Figure 2, I–N, and Supplemental Figure 2, B–E). Together, these findings reinforce that TAM-derived GPNMB is critical for regulating GSC glycolysis.

### TAM-derived GPNMB promotes GSC self-renewal.

**Figure 3:**
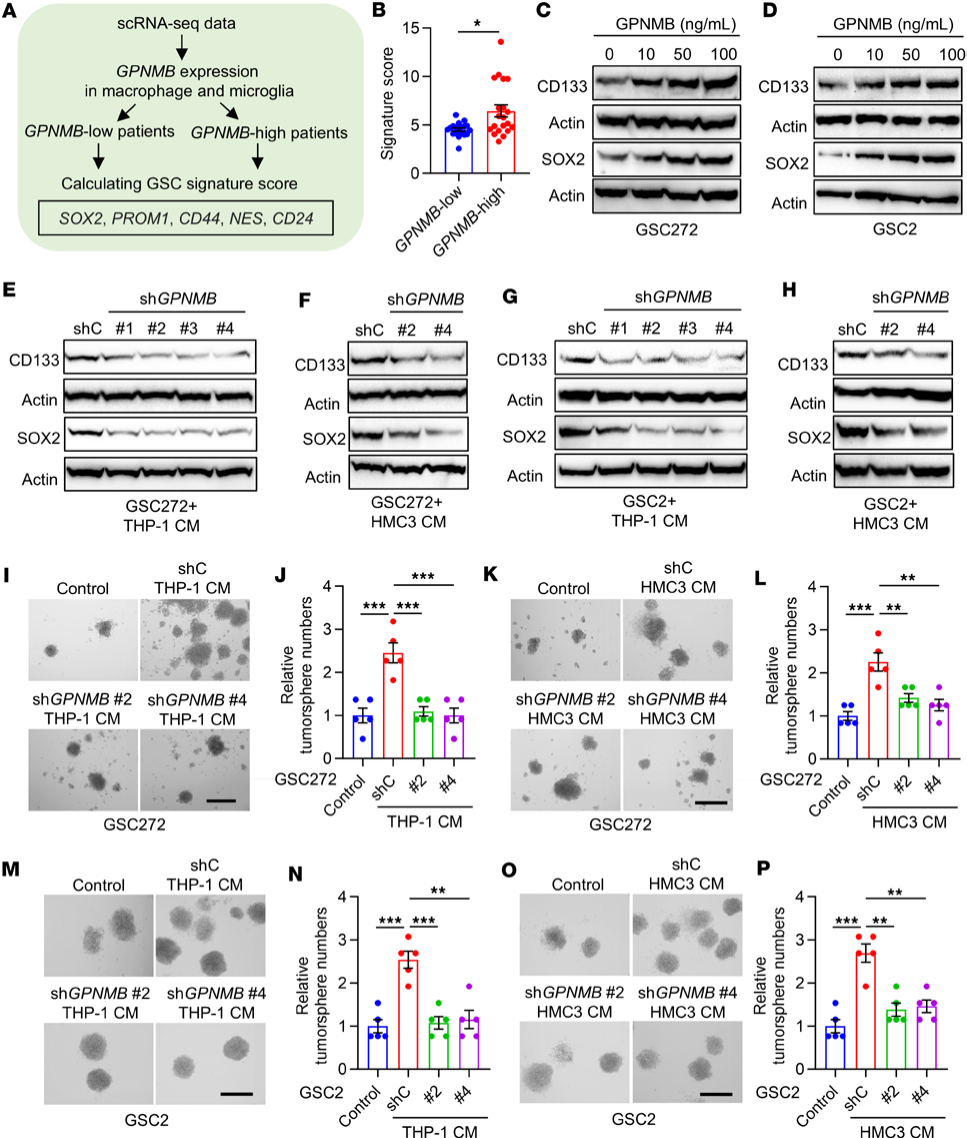
GPNMB promotes the stemness of GSCs. (**A**) Strategy to cluster GBM samples into *GPNMB*-low and *GPNMB*-high subgroups based on 4 published single-cell RNA-Seq (scRNA-Seq) datasets (GSE131928, GSE148842, GSE89567, and EGAS00001004422). (**B**) GSC signature score in *GPNMB*-low (*n* = 19) and *GPNMB*-high (*n* = 20) subgroups based on the above 4 scRNA-Seq datasets. Student’s *t* test. (**C** and **D**) Immunoblots for CD133 and SOX2 in lysates of GSC272 (**C**) and GSC2 (**D**) cells treated with GPNMB recombinant protein at the different indicated concentrations for 24 hours. (**E** and **F**) Immunoblots for CD133 and SOX2 in lysates of GSC272 cells treated with the conditioned media (CM) of THP-1 macrophages (**E**) and HMC3 microglia (**F**) expressing shRNA control (shC) or *GPNMB* shRNA (sh*GPNMB*) for 24 hours. (**G** and **H**) Immunoblots for CD133 and SOX2 in lysates of GSC2 cells treated with the CM of THP-1 macrophages (**G**) and HMC3 microglia (**H**) expressing shC and sh*GPNMB* for 24 hours. (**I**–**L**) Representative images and quantification of tumorspheres of GSC272 cells treated with the CM of THP-1 macrophages (**I** and **J**) or HMC3 microglia (**K** and **L**) expressing shC and sh*GPNMB* for 2 weeks. Scale bar: 200 μm. *n* = 5 independent samples. One-way ANOVA test. (**M**–**P**) Representative images and quantification of tumorspheres of GSC2 cells treated with the CM of THP-1 macrophages (**M** and **N**) or HMC3 microglia (**O** and **P**) expressing shC and sh*GPNMB* for 2 weeks. Scale bar: 200 μm. *n* = 5 independent samples. One-way ANOVA test. *P < 0.05, **P < 0.01, ***P < 0.001.

Given the important connection between glycolysis and stemness in cancer cells (38–40), we next explored the potential role of GPNMB in regulating GSC self-renewal. To this end, GBM samples from 4 published scRNA-Seq datasets (41–44) were clustered into *GPNMB*-low and *GPNMB*-high subgroups according to the expression level of *GPNMB* in TAMs (Figure 3A). We found that patients with GBM in *GPNMB*-high subgroup exhibited a higher GSC signature (45) compared with those in *GPNMB*-low subgroup (Figure 3B). To investigate the role of GPNMB in promoting GSC self-renewal through experimentation, we added recombinant GPNMB protein to patient-derived GSCs, including GSC272 and GSC2 cells. Immunoblotting results demonstrated that GPNMB protein treatment enhanced the expression of stemness-associated factors CD133 and SOX2 (Figure 3, C and D) as well as GSC self-renewal (Supplemental Figure 3, A–D) and proliferation (Supplemental Figure 3, E and F). To confirm the role of TAM-derived GPNMB in this process, we depleted GPNMB in human THP-1 macrophages and HMC3 microglia (Supplemental Figure 2, B and C). Compared with the CM from shC cells, CM from GPNMB-depleted THP-1 macrophages and HMC3 microglia decreased the expression of CD133 and SOX2 (Figure 3, E–H), inhibited self-renewal (Figure 3, I–P), and impaired proliferation (Supplemental Figure 3, G and H) in GSC272 and GSC2 cells. In summary, these data highlight that TAM-derived GPNMB is essential for GSC self-renewal.

### GPNMB promotes GSC glycolysis and self-renewal by activating the PYK2/RSK2 signaling axis.

**Figure 4:**
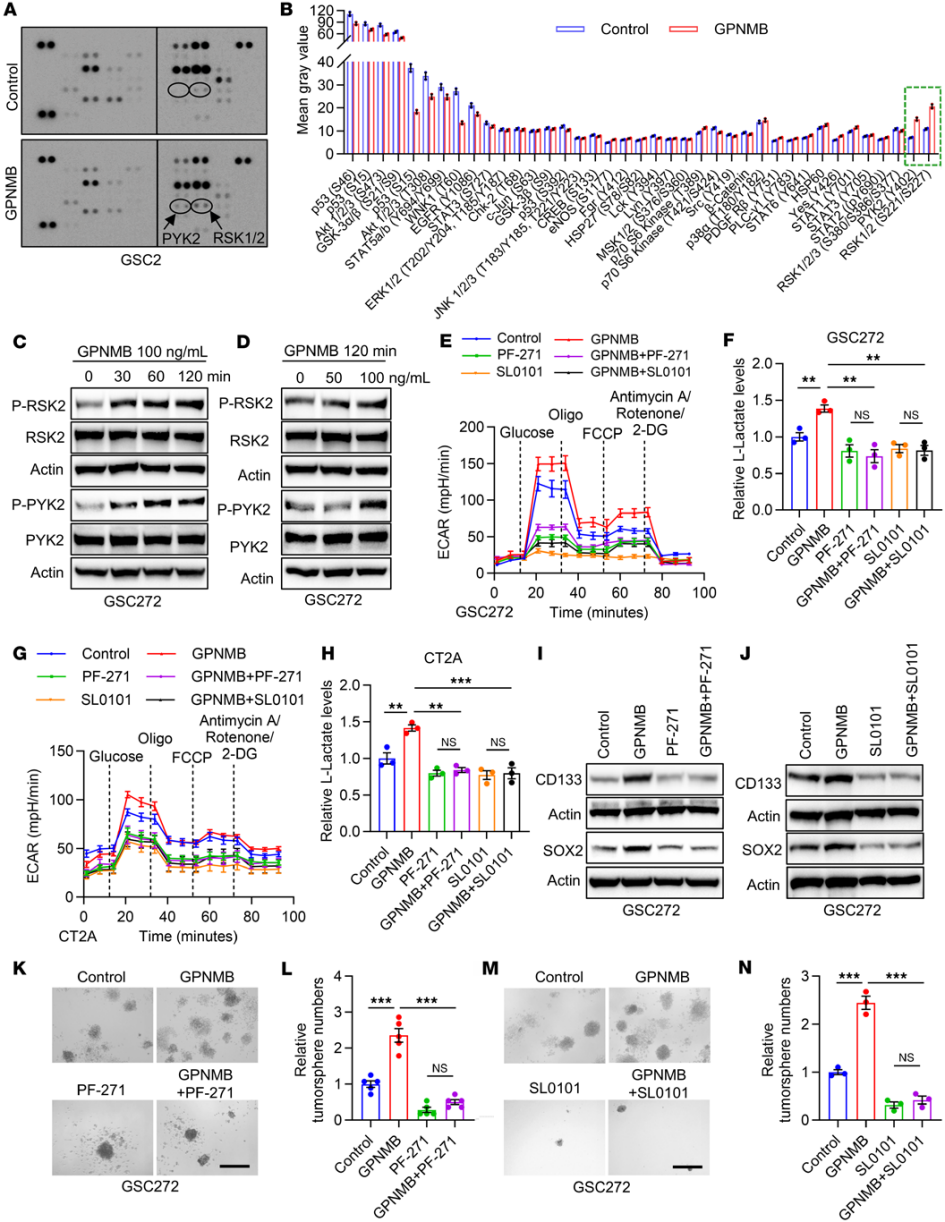
GPNMB promotes GSC glycolysis and stemness by activating PYK2/RSK2 axis. (**A** and **B**) Representative images (**A**) and quantification (**B**) of human phospho-kinases in GSC2 cells treated with or without GPNMB recombinant protein (100 ng/mL) for 1 hour. Affected kinases are indicated. (**C** and **D**) Immunoblots for P-RSK2, RSK2, P-PYK2, and PYK2 in lysates of GSC272 cells treated with GPNMB recombinant protein at the indicated concentrations and time points. (**E**) Extracellular acidification rate (ECAR) of GSC272 cells treated with GPNMB recombinant protein (100 ng/mL) in the presence or absence of PYK2 inhibitor PF-271 (15 nM) or RSK1/2 inhibitor SL0101 (100 μM) for 24 hours. *n* = 6 independent samples. (**F**) Relative L-lactate levels in GSC272 cells treated with GPNMB recombinant protein (100 ng/mL) in the presence or absence of PF-271 (15 nM) or SL0101 (100 μM) for 24 hours. *n* = 3 independent samples. One-way ANOVA test. (**G**) ECAR of CT2A cells treated with GPNMB recombinant protein (100 ng/mL) in the presence or absence of PF-271 (15 nM) or SL0101 (100 μM) for 24 hours. *n* = 6 independent samples. (**H**) Relative L-lactate levels in CT2A cells treated with GPNMB recombinant protein (100 ng/mL) in the presence or absence of PF-271 (15 nM) or SL0101 (100 μM) for 24 hours. *n* = 3 independent samples. One-way ANOVA test. (**I** and **J**) Immunoblots for CD133 and SOX2 in lysates of GSC272 cells treated with GPNMB recombinant protein (100 ng/mL) in the presence or absence of PF-271 (15 nM, **I**) or SL0101 (100 μM, **J**) for 24 hours. (**K**–**N**) Tumorsphere formation assays of GSC272 cells treated with GPNMB recombinant protein (100 ng/mL) in the presence or absence of PF-271 (15 nM, **K** and **L)** or SL0101 (100 μM, **M** and **N**) for 2 weeks. Scale bar: 200 μm. *n* = 3–5 independent samples. One-way ANOVA test. **P < 0.01, ***P < 0.001, n.s., not significant (*P* > 0.05).

To identify the downstream signals that can mediate GPNMB-induced GSC glycolysis and stemness, we performed an unbiased study using a human phospho-kinase antibody array in GSC2 cells treated with or without GPNMB protein. The results showed that PYK2 and RSK1/2 were activated in association with GPNMB protein treatment (Figure 4, A and B). Immunoblotting confirmed that the phosphorylation of PYK2 and RSK2 in GSC2 and GSC272 cells was increased upon the treatment with GPNMB protein (Figure 4, C and D, and Supplemental Figure 4, A and B). To investigate whether PYK2 and RSK2 are required for GPNMB’s effects on GSCs, we performed glycolysis assays, with results showing that pharmacologic inhibition of PYK2 with PF-00562271 (PF-271) or RSK2 with SL 0101-1 (SL0101) abolished GPNMB-induced ECAR and lactate upregulation in GSC272, GSC2, and CT2A cells (Figure 4, E–H, and Supplemental Figure 4C). Next, the stemness-related assays demonstrated that treatment of GSCs with PF-271 or SL0101 negated the expression of CD133 and SOX2 (Figure 4, I and J), self-renewal (Figure 4, K–N, and Supplemental Figure 4, D–G), and proliferation (Supplemental Figure 4, H and I) effects of elevating cellular GPNMB. To reveal the relationship between PYK2 and RSK2 in GSCs, GPNMB recombinant protein-conditioned GSCs were treated with or without PF-271 or SL0101. Immunoblotting results demonstrated that GPNMB-induced RSK2 activation was abolished by PYK2 inhibition (Supplemental Figure 4, J and K). However, RSK2 inhibition had no effect on GPNMB-induced PYK2 activation (Supplemental Figure 4, L and M). Together, these findings support a critical role for the PYK2/RSK2 signaling axis in mediating GPNMB-directed GSC glycolysis and self-renewal.

### CD44 is essential for GPNMB-induced PYK2/RSK2 axis activation, glycolysis, and self-renewal in GSCs.

**Figure 5:**
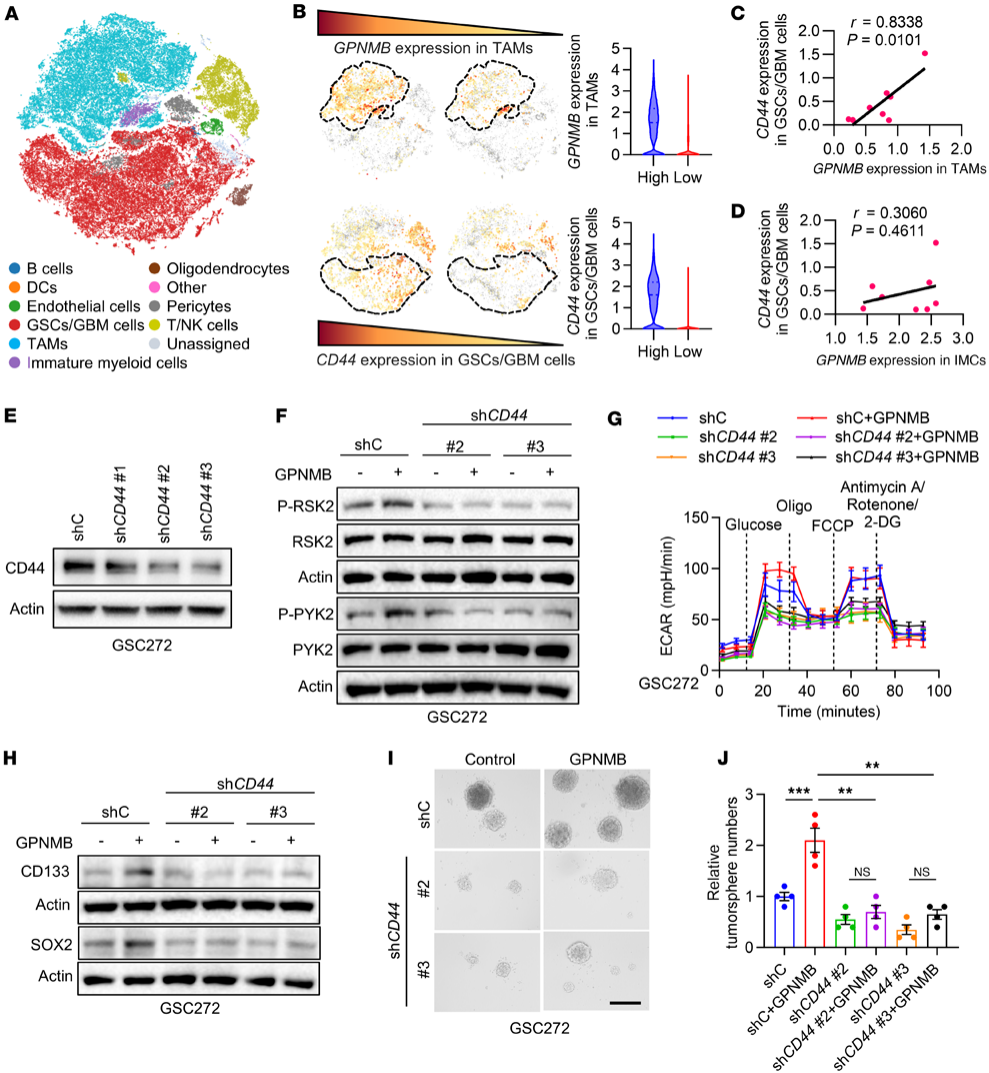
GPNMB induces GSC glycolysis and stemness through CD44. (**A**) T-distributed stochastic neighbor embedding (t-SNE) dimensional reduction of cancer cells and immune cells from GBM tumor samples based on single-cell RNA-Seq (scRNA-Seq) dataset (GSE182109). (**B**) Pattern representing single-cell gene expression (left) and quantification (right) of *GPNMB* in TAMs and *CD44* in GSCs/GBM cells based on scRNA-Seq dataset (GSE182109). (**C**) Relationship between *GPNMB* expression in TAMs and *CD44* expression in GSCs/GBM cells based on scRNA-Seq dataset (GSE182109). *P* and *r* values are shown. Pearson’s test. (**D**) Relationship between *GPNMB* expression in IMCs and *CD44* expression in GSCs/GBM cells based on scRNA-Seq dataset (GSE182109). *P* and *r* values are shown. Pearson’s test. (**E**) Immunoblots for CD44 in lysates of GSC272 cells expressing shRNA control (shC) or *CD44* shRNA (sh*CD44*). (**F**) Immunoblots for P-RSK2, RSK2, P-PYK2, and PYK2 in lysates of GSC272 cells expressing shC and sh*CD44* treated with or without GPNMB recombinant protein (100 ng/mL) for 2 hours. (**G**) Extracellular acidification rate (ECAR) of GSC272 cells expressing shC and sh*CD44* treated with or without GPNMB recombinant protein (100 ng/mL) for 24 hours. ECAR was obtained from the Seahorse experiments, and glucose was added at the indicated time point. *n* = 3 independent samples. (**H**) Immunoblots for CD133 and SOX2 in lysates of GSC272 cells expressing shC and sh*CD44* treated with or without GPNMB recombinant protein (100 ng/mL) for 24 hours. (**I** and **J**) Representative images (**I**) and quantification (**J**) of tumorspheres of GSC272 cells expressing shC and sh*CD44* treated with or without GPNMB recombinant protein (100 ng/mL) for 2 weeks. Scale bar: 200 μm. *n* = 4 independent samples. One-way ANOVA test. **P < 0.01, ***P < 0.001, n.s., not significant (*P* > 0.05).

Since previous studies have shown that CD44 is the functional receptor for GPNMB (46), we hypothesized that GPNMB-induced PYK2/RSK2 activation, glycolysis, and self-renewal in GSCs might be regulated by CD44. The scRNA-Seq data analysis in tumors from patients with GBM (47) demonstrated that *CD44* expression in GSCs/GBM cells positively correlated with *GPNMB* expression level in TAMs but not in IMCs (Figure 5, A–D), suggesting a potential ligand-receptor interaction between TAMs and GSCs/GBM cells. Immunoblotting and glycolysis assays demonstrated that shRNA-mediated depletion of CD44 (Figure 5E) abolished GPNMB-triggered PYK2 and RSK2 activation (Figure 5F) and ECAR (Figure 5G) in GSC272 cells. Functionally, these effects were accompanied by reduced stemness, as indicated by the impaired CD133 and SOX2 expression (Figure 5H) and tumorsphere formation (Figure 5, I and J), upon CD44 depletion in GPNMB-treated GSC272 cells. In summary, these findings support the role of CD44 in mediating GPNMB-induced GSC glycolysis and self-renewal through the PYK2/RSK2 signaling axis.

### Inhibition of GPNMB-mediated GSC-TAM crosstalk extends survival in GBM models.

**Figure 6:**
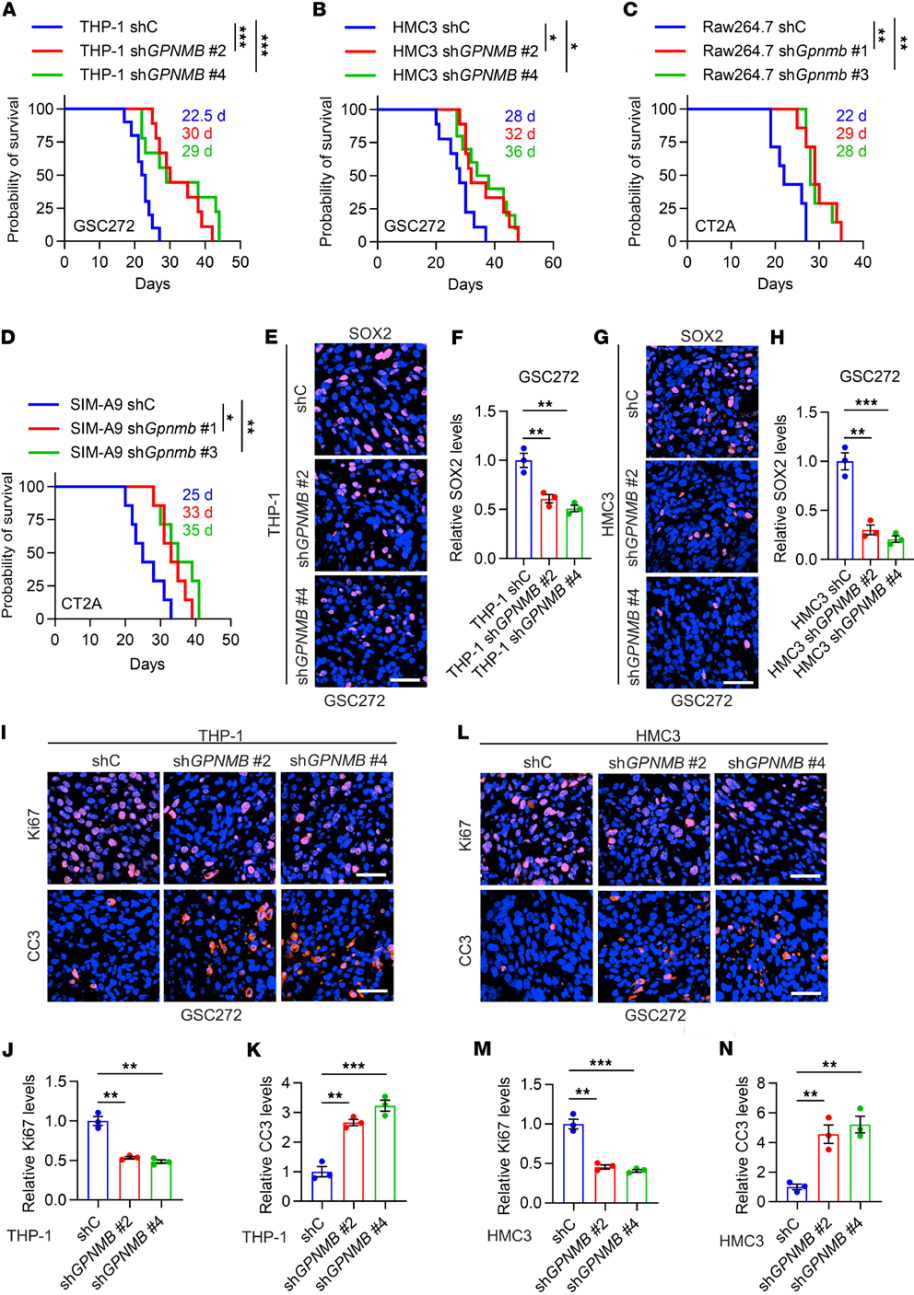
Inhibition of GPNMB in microglia and macrophages inhibits tumor growth and extends survival in various GBM models. (**A** and **B**) Survival curves of nude mice coimplanted with GSC272 cells (1 × 10^5^ cells/mouse) and THP-1 macrophages (1 × 10^5^ cells/mouse, **A**) or HMC3 microglia (1 × 10^5^ cells/mouse, **B**) expressing shRNA control (shC) or *GPNMB* shRNA (sh*GPNMB*). *n* = 9–10 mice per group. Median survival days are shown. Log-rank test. (**C** and **D**) Survival curves of C57BL/6 mice coimplanted with CT2A cells (1 × 10^4^ cells/mouse) and Raw264.7 macrophages (1 × 10^4^ cells/mouse, **C**) or SIM-A9 microglia (1 × 10^4^ cells/mouse, **D**) expressing shC or sh*Gpnmb*. *n* = 7 mice per group. Median survival days are shown. Log-rank test. (**E**–**H**) Representative images and quantification of immunofluorescence staining of SOX2 in tumors from the brains of nude mice intracranially coimplanted with GSC272 cells and THP-1 macrophages (**E** and **F**) or HMC3 microglia (**G** and **H**) expressing shC or sh*GPNMB*. Scale bar: 50 μm. *n* = 3 independent samples. One-way ANOVA test. (**I**–**K**) Representative images (**I**) and quantification (**J** and **K**) of immunofluorescence staining of Ki67 (**I** and **J**) and CC3 (**I** and **K**) in tumors from the brains of nude mice intracranially coimplanted with GSC272 cells and THP-1 macrophages expressing shC or sh*GPNMB*. Scale bar: 50 μm. *n* = 3 independent samples. One-way ANOVA test. (**L**–**N**) Representative images (**L**) and quantification (**M** and **N**) of immunofluorescence staining of Ki67 (**L** and **M**) and CC3 (**L** and **N**) in tumors from the brains of nude mice intracranially coimplanted with GSC272 cells and HMC3 microglia expressing shC or sh*GPNMB*. Scale bar: 50 μm. *n* = 3 independent samples. One-way ANOVA test. *P < 0.05, **P < 0.01, ***P < 0.001.

To further investigate the in vivo effect of GPNMB-directed GSC-TAM bidirectional communication using GBM mouse models, we utilized shRNA knockdown system to deplete GPNMB in macrophages and microglia (Supplemental Figure 2, B–E) and then implanted them together with human and mouse GSC/GBM cells into nude and C57BL/6 mice, respectively. In human GSC272 cells PDX model, we found that depletion of GPNMB in THP-1 macrophages or HMC3 microglia significantly prolonged the survival of tumor-bearing mice (Figure 6, A and B). Similarly, depletion of GPNMB in Raw264.7 macrophages or SIM-A9 microglia significantly extended survival of CT2A tumor-bearing mice (Figure 6, C and D). To confirm whether GSC stemness is involved in the impaired tumor progression upon GPNMB depletion in macrophages and microglia, we performed immunofluorescence staining for SOX2 in shC and sh*GPNMB* tumors. The results showed that depletion of GPNMB in macrophages and microglia downregulated GSC stemness, as indicated by the reduced SOX2^+^ cells in GPNMB-depleted tumors (Figure 6, E–H). Immunofluorescence for Ki67 and cleaved caspase-3 (CC3) confirmed that proliferation was dramatically reduced, whereas apoptosis was increased in GSC272 cells tumors with macrophages or microglia harboring GPNMB depletion (Figure 6, I–N). Together with the data showing that GPNMB is preferentially expressed by TAMs in mouse and human GBM (Figure 1), these findings support the importance of TAM-derived GPNMB in promoting GSC stemness and GBM progression in vivo.

### GPNMB correlates with TAM abundance and GSC stemness in human GBM.

**Figure 7:**
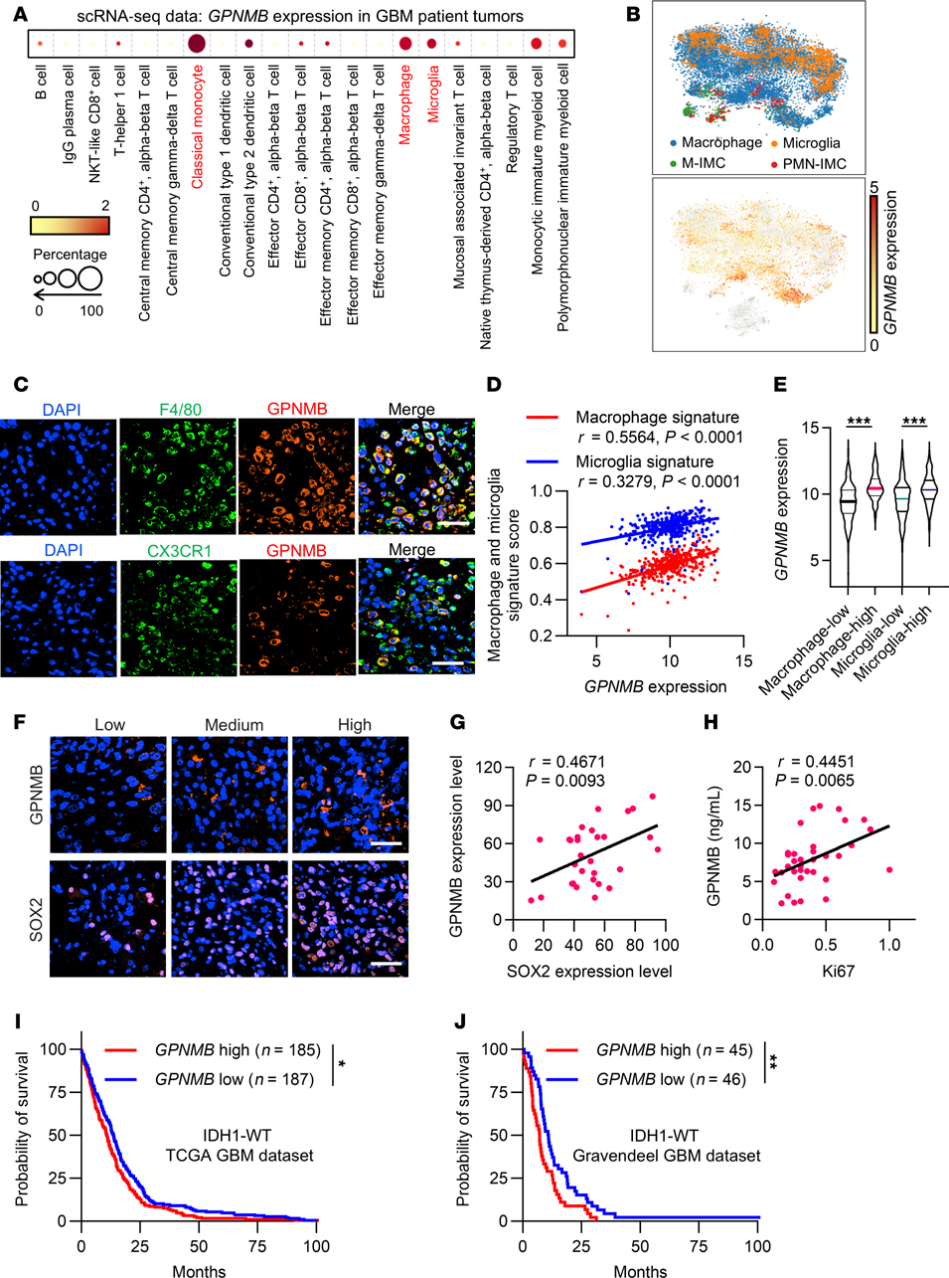
GPNMB is a prognostic biomarker correlating with TAM prominence and GSC stemness in human GBM. (**A**) GPNMB expression levels in different types of immune cells from GBM tumors based on single-cell RNA-Seq (scRNA-Seq) dataset (EGAS00001004871). (**B**) Pattern representing single-cell gene expression of *GPNMB* in macrophages, microglia, M-IMCs, and PNM-IMCs from GBM tumors based on scRNA-Seq dataset (EGAS00001004871). (**C**) Coimmunofluorescence staining for GPNMB (red) and F4/80 (macrophage marker, green) or CX3CR1 (microglia marker, green) in human GBM tumor samples. Scale bar: 50 μm. (**D**) Correlation between *GPNMB* expression and macrophage/microglia signature scores in IDH1-WT TCGA samples from patients with GBM. *P* and *r* values are shown. Pearson’s test. (**E**) The expression of *GPNMB* in macrophage-high (*n* = 185) and macrophage-low (*n* = 187) as well as microglia-high (*n* = 185) and microglia-low (*n* = 187) groups of IDH1-WT TCGA samples from patients with GBM. Student’s *t* test. (**F** and **G**) Representative images (**F**) and correlation quantification analysis (**G**) between GPNMB and SOX2 expression in human GBM tumor samples (*n* = 30) based on immunofluorescence staining. Scale bar: 50 μm. *P* and *r* values are shown. Pearson’s test. (**H**) Relationship between histological Ki67 score in tumors and GPNMB expression level in the plasma of patients with GBM (*n* = 36). *P* and *r* values are shown. Pearson’s test. (I and J) Survival curves of patients with GBM with high and low GPNMB expression in IDH1-WT TCGA (I) and Gravendeel (J) GBM datasets from GlioVis (https://gliovis.bioinfo.cnio.es/). Log-rank test. *P < 0.05, **P < 0.01, ***P < 0.001.

To confirm the clinical relevance of our findings, we performed analysis of scRNA-Seq data of tumors from patients with GBM (37), showing that *GPNMB* was preferentially expressed in classical monocytes, macrophages, and microglia (Figure 7, A and B, and Supplemental Figure 5A). In addition, immunofluorescence staining in tumors from patients with GBM confirmed that GPNMB was mainly expressed in F4/80^+^ macrophages and CX3CR1^+^ microglia (Figure 7C). Then, we calculated macrophage and microglia gene signatures (48, 49) in IDH1-WT samples from patients with GBM from The Cancer Genome Atlas (TCGA), with results showing that GPNMB expression positively correlated with macrophage and microglia signatures (Figure 7D). Using macrophage and microglia clustering in IDH1-WT TCGA GBM tumors (Supplemental Figure 5, B and C), we found that patients in the macrophage-high and microglia-high group exhibited poorer survival (Supplemental Figure 5, D and E) and higher GPNMB expression (Figure 7E) compared with patients in the macrophage-low and microglia-low group, respectively. To further confirm the connection between GPNMB and GSC stemness, we performed validation assays using tumor and plasma samples from patients with GBM and found that GPNMB levels in tumors and plasma from patients with GBM positively correlated with their intratumoral SOX2 (Figure 7, F and G) and Ki67 (Figure 7H) expression, respectively. In TCGA and Gravendeel GBM datasets (50, 51), *GPNMB* expression was negatively correlated with patient overall survival in IDH1-WT (Figure 7, I and J), but not IDH1-mutant (Supplemental Figure 5, F and G), GBM. Together, these findings from human GBM support the conclusion that GPNMB is highly expressed in TAMs and TAM-derived GPNMB contributes to GSC self-renewal and GBM progression.

## Discussion

In this study, we uncover GPNMB as a key factor mediating the bidirectional interaction between GSCs and TAMs in GBM. Specifically, GSCs educate macrophages and microglia to preferentially express and secrete GPNMB in the GBM TME, which, in turn, promotes tumor progression. Mechanistically, TAM-derived GPNMB binds to its receptor CD44 on GSCs to promote glycolysis and stemness via activating the PYK2/RSK2 signaling axis. Depletion of GPNMB in TAMs results in increased survival in GBM mouse models, underscoring the potential therapeutic value of targeting TAM-derived GPNMB for GBM treatment.

GPNMB is a glycosylated type I transmembrane protein and is localized on the cell surface or stored in endosomes/lysosomes (23, 52). It plays an important role in diverse physiological processes, given it is widely expressed in various cell types and tissues, such as osteoblasts and osteoclasts in the bone (20, 53) and melanocytes, keratinocytes, and Langerhans cells in the skin (54, 55). GPNMB is markedly upregulated in cancer cells (25, 56–59) or the TME (30, 32, 46, 60, 61), where it exhibits a protumor function through a paracrine and/or autocrine mechanism. In GBM, GPNMB has been found to be increased at both mRNA and protein levels in patient tumors compared with normal brain samples (25). In identifying key TAM-derived factors mediating the GSC-TAM bidirectional communication, we observed that GPNMB is predominantly expressed and secreted by TAMs in GBM tumors, consistent with a most recent report (30). We also found that GSCs can educate macrophages and microglia to express GPNMB in the GBM TME; however, further studies are needed to reveal the detailed mechanisms underlying this process. We also observed that GPNMB can be expressed by IMCs within GBM TME, although at significantly lower levels compared with TAMs. Given that IMCs can differentiate into TAMs and influence macrophage polarization within the TME (62), IMC-derived GPNMB may also contribute to GBM progression through distinct or overlapping mechanisms.

Increasing evidence demonstrates a potential role of TAM-derived GPNMB in GBM progression by affecting GBM cell proliferation and mesenchymal transition as well as immune cell infiltration and activation (30, 31). In our study, we established that TAM-derived GPNMB drives GBM progression via promoting GSC self-renewal (a key GBM hallmark), generating a critical TAM-GSC bidirectional interaction. Mounting evidence points to an essential role of the bidirectional interaction between GSCs and TAMs in GBM progression and therapy (6, 10). Specifically, our recent findings have demonstrated that GSC/GBM cell–derived cytokines (e.g., LOX, OLFML3, LGMN, TFPI2, and CCL2/CCL7) are important to generate a context-dependent GSC-TAM bidirectional interaction, inducing a protumor TME (13–17). The results from this study not only reinforce the importance of TAM-GSC bidirectional communication in GBM biology, but also highlight the role of TAM-derived factor (GPNMB) in mediating this bidirectional communication.

To reveal the role of TAM-derived GPNMB in GBM cell biology, we performed scRNA-Seq analysis and observed that glycolysis signature is enriched in GBM tumors harboring high GPNMB expression in TAMs. These data combined functional validations reveal the substantial function of TAM-derived GPNMB in regulating glycolysis in GBM cells, a conclusion that is supported by the findings obtained from ovarian cancer (63). Similarly, TAM-derived IL-6 can facilitate PDPK1-mediated glycolysis in GBM cells to promote tumor growth (64). In addition to glycolysis, TAMs can affect GBM progression via other metabolic processes. For example, GPNMB-high macrophage subsets in the GBM TME (termed lipid-laden macrophages, can transfer myelin-derived lipids to GBM cells, fueling cancer cell proliferation and recurrence (65). Together with the results in the current study, we propose that TAM-derived GPNMB serves as a key factor promoting tumor progression by regulating GBM cell metabolism.

Given the connection between glycolysis and stemness in cancer cells (38–40) and previous findings obtained from pancreatic cancer and fibrosarcoma, showing that TAM-derived GPNMB can promote cancer cell stemness (46), we further observed that GPNMB is a key factor driving GSC self-renewal. In dissecting the molecular mechanism underlying GPNMB’s role in promoting GSC self-renewal, we performed an unbiased experiment using a phospho-kinase antibody array followed by immunoblotting validations and confirmed that GPNMB activates the PYK2/RSK2 signaling pathway. These findings reinforce the importance of the PYK2 signaling in GSC stemness, which is consistent with previous work showing that PYK2 is important for breast cancer cell stemness maintenance (66–68), and highlight RSK2 as a downstream of PYK2 responsible for GPNMB-triggered GSC self-renewal. Previous work in breast cancer cells has shown that PYK2 can activate STAT3 (67), a transcription factor that is important for GSC maintenance and self-renewal (17, 69). Therefore, it will be very interesting to investigate the relationship between RSK2 and STAT3 in GSCs, which is also supported by the evidence in GBM cells showing that RSK2 and STAT3 are connected (70). On the other hand, our recent work has shown that PYK2 is required for LOX-induced macrophage infiltration in GBM (13). These findings suggest that GPNMB might exhibit a cell-autonomous effect to trigger macrophage infiltration via activating PYK2 signaling, which could further increase GPNMB expression to support the bidirectional interaction between TAMs and GSCs by triggering a positive feedback loop. Together, these findings point out the importance of the PYK2/RSK2 signaling axis responsible for GPNMB-triggered GSC glycolysis and self-renewal and highlight the potential of testing PYK2 and RSK2 inhibitors in patients with GBM.

In exploring the connection between GPNMB and the PYK2/RSK2 signaling axis in GSCs, we observed that the expression of CD44 in GSCs/GBM cells is positively correlated with GPNMB expression in TAMs of tumors from patients with GBM. This finding is reinforced by our functional studies showing that depletion of CD44 abolished GPNMB-triggered PYK2/RSK2 signaling activation, glycolysis, and self-renewal in GSCs and by previous studies supporting the critical role of CD44 in regulation of GSC stemness (71, 72). Extending the findings in nonbrain tumor models showing that GPNMB binds to its receptor CD44 on cancer cells to promote stemness via upregulating IL-33 (46), our studies reveal an additional mechanism: that CD44 functions as a GPNMB receptor and mediates GPNMB’s stemness-promoting effect via activating the PYK2/RSK2 signaling pathway. These findings highlight that CD44 serves as a functional GPNMB receptor on GSCs and provide biological insights to TAM-GSC bidirectional communication.

In summary, our work reveals that GSC-educated TAMs are the main resource of GPNMB in GBM, where it promotes GSC glycolysis and stemness via activating the CD44-PYK2/RSK2 signaling axis. The identification of GPNMB as the key factor mediating TAM-GSC bidirectional communication, combined with our findings showing antitumor effects resulting from TAM depletion of GPNMB in GBM mouse models, indicates an effective therapeutic intervention strategy of targeting TAM-GSC bidirectional communication and encourages the development of therapeutic agents targeting GPNMB-expressing TAMs in patients with GBM. It should be noted that this study focused on TAM-derived GPNMB in GSC biology, while its role on GBM cells remains to be explored. Moreover, the coinjection of GSCs and GPNMB-depleted TAMs into mouse brains may not fully recapitulate TAM dynamics in vivo. Developing TAM-specific GPNMB-knockout mice would help address this limitation. Finally, although GPNMB blockade modestly extends the survival of tumor-bearing mice, combining GPNMB inhibition with standard-of-care therapies (e.g., radiotherapy and chemotherapy) or targeting the downstream PYK2/RSK2 signaling axis may enhance translational relevance and therapeutic efficacy.

## Methods

### Sex as a biological variable.

Sex was not considered as a biological variable in this study.

### Cell culture.

THP-1 and Raw264.7 macrophage cell lines were cultured in RPMI 1640 medium (Gibco, 11875093). HMC3 and SIM-A9 microglia were cultured in Eagle’s Minimum Essential Medium (ATCC, 30-2003) and Dulbecco’s Modified Eagle Medium–Ham’s F12 medium (Gibco, 10565-018), respectively. 293T cell line was cultured in Dulbecco’s Modified Eagle Medium (Gibco, 11995-065). All these cell lines were cultured in indicated medium containing 10% FBS (Fisher Scientific, 16140071) and 1:100 antibiotic-antimycotic (Gibco, 15140-122) and were purchased from the ATCC. Raw264.7 and THP-1 cells were differentiated into macrophages by adding 200 ng/mL phorbol 12-myristate 13-acetate (Sigma-Aldrich, P8139) for 24 hours before further experiments were conducted (73). For stemness maintenance, CT2A cells were cultured in neural stem cell proliferation media (Millipore, SCM005) containing 20 ng/mL epidermal growth factor (PeproTech, AF-100-15) and basic fibroblast growth factor (PeproTech, 100-18B). Human patient-derived GSC2 and GSC272 cells were gifted by Frederick F. Lang (Brain Tumor Center, The University of Texas MD Anderson Cancer Center, Houston, Texas, USA). Mouse GBM tumor–derived GSC line QPP7 was provided by Jian Hu (The University of Texas MD Anderson Cancer Center). All human and mouse GSCs were cultured in neural stem cell proliferation medium containing 20 ng/mL epidermal growth factor and basic fibroblast growth factor. All cells were confirmed to be mycoplasma-free and were maintained at 37°C and 5% CO_2_. CM were collected from GSCs, or number-matched shC and *GPNMB* shRNA knockdown cells after culture for another 24 hours in growth factor–free or FBS-free culture medium.

### Mice and intracranial xenograft tumor models.

Female C57BL/6 (catalog 0000664) and nude (catalog 007850) mice at 4 to 6 weeks of age were purchased from The Jackson Laboratory. All animals were grouped by 5 mice per cage and maintained in IVC System (Lab Products, LLC) for a week before the experiment. GSC272 or CT2A cells were incubated with the CM of shC and sh*GPNMB* macrophages or microglia for 12 hours. Then, the treated GSC272 cells or CT2A cells were mixed with macrophages or microglia expressing shC or sh*GPNMB* by a 1:1 ratio. The intracranial xenograft tumor models were established as described previously (13, 14, 17). In brief, mice were anesthetized using isoflurane through IMPAC6 Anesthesia System. Then, a dental drill was used to open a small hole (1.2 mm anterior and 3.0 mm lateral to the bregma) in the skulls of mice. Mice were placed into the stereotactic apparatus, and 5 μL mixed cells in growth factor–free or FBS-free culture medium were injected into the right caudate nucleus 3 mm below the surface of the brain using a 10 μL Hamilton syringe with an unbeveled 30-gauge needle. The incision was closed using Vetbond glue (3M Science). Meloxicam (20 mg/kg, daily) was subcutaneously injected for pain relief for 3 days after the surgery. Then, mice were monitored for recording survival. Mice with neurological deficits or moribund appearance were sacrificed according to the IACUC protocol. At the end of the experiment, the brains of mice were isolated, either fixed in 4% paraformaldehyde after transcardiac perfusion with PBS for optimal cutting temperature–cryosectioning or processed to isolate tumor-derived immune cells for flow cytometry analysis.

### Human samples.

Deidentified human plasma (*n* = 40) and tumor samples (*n* = 30) from patients with GBM were obtained from the Northwestern Central Nervous System Tissue Bank (NSTB, Chicago, USA). Patients were diagnosed according to the WHO diagnostic criteria by an in-house neuropathologist at NSTB. Detailed patient information is provided in Supplemental Table 1. The healthy control plasma (*n* = 8) was purchased from Solomon Park Company.

### Computational analysis of human GBM datasets.

For analysis of human GBM samples, we downloaded the gene expression data from TCGA-GBM, TCGA-LGG, Rembrandt (36), and Gravendeel (51) datasets from GlioVis (http://https://gliovis.bioinfo.cnio.es/). The enrichment scores of infiltrating macrophages and microglia in each sample were investigated by using the single-sample GSEA (ssGSEA) based on the list of 33 human macrophage signature genes (48) and the 9 human microglia signature genes (49), respectively. This analysis was conducted by using ssGSEA algorithm via the “GSVA” package in R software (v.4.3.3).

The single-cell sequencing data of tumors from patients with GBM were downloaded and analyzed by using the BBrowser BioTuring under Talk2Data platform based on established methodology (74). Data from public repositories were integrated and used in this study, including EGAS00001004871 (37) and EGAS00001004422 (44) from the European Genome-phenome Archive (EGA) and GSE131928 (41), GSE148842 (42), GSE89567 (43) and GSE182109 (47) from the Gene Expression Omnibus (GEO). Based on *GPNMB* expression in macrophages and microglia, patients were regrouped into *GPNMB*-low and *GPNMB*-high subgroups. Cancer cells or GSCs from each group were selected for GSEA analysis.

### Plasmids and viral transfections.

shRNAs targeting human *GPNMB* and *CD44* and mouse *Gpnmb* in the pLKO.1 vector (Sigma-Aldrich, SHC001) were used. Lentiviral particles were generated as we described previously (14, 17). In brief, 8 μg of the shRNA plasmid, 4 μg of the psPAX2 plasmid (Addgene, 12260), and 2 μg of the pMD2.G plasmid (Addgene, 12259) were transfected into 293T cells plated in 100 mm dishes using Lipofectamine 2000 (Invitrogen, 13778150). Supernatant with lentiviral particles was collected and filtered at 48 and 72 hours after transfection. Cells were infected with viral supernatants containing 10 μg/mL polybrene (Millipore, TR-1003-G). After 48 hours, cells were selected by puromycin-containing (10 μg/mL; Millipore, 540411) medium for 1 week. Then, cells were cultured in low-puromycin-containing (2 μg/mL) medium to keep selecting pressure. The knockdown efficiency was tested for the expression of GPNMB and CD44 using immunoblots. The following human and mouse shRNA sequences were used in this study, *GPNMB*, #1, TRCN0000178982; #2, TRCN0000151637; #3, TRCN0000154721; and #4, TRCN0000179643; *CD44*, #1, TRCN0000308110; #2, TRCN0000296191; and #3, TRCN0000289233; and *Gpnmb*, #1, TRCN0000294593; #2, TRCN0000307370; #3, TRCN0000294525; and #4, TRCN0000294523.

### Immunofluorescence.

Immunofluorescence was conducted following a previously outlined standard protocol (14, 17). Briefly, cryosection slides were left at room temperature for 30 minutes and then fixed in 10% paraformaldehyde for another 30 minutes before permeabilization. Subsequently, 0.25% Triton X-100 in PBS was applied for 30 minutes at room temperature to facilitate cell membrane permeabilization. Following 3 PBS washes, the sections were blocked with 5% goat serum for 30 minutes. Primary antibody incubation was carried out for 1 hour at room temperature and then overnight at 4°C. Unbound primary antibodies were removed by three 3-minute PBS washes, and secondary antibody cocktails were prepared and applied to the sections for 1-hour incubation. Cell nuclei were counterstained using DAPI/anti-fade mounting medium (Vector Laboratories, H-1200-10). Immunofluorescence images were captured using the Nikon AX/AX R Confocal Microscope System equipped with an apo 60 1.40 Oil 160/0.17 objective. The protein signal’s relative intensity was quantified using ImageJ (NIH). Antibodies specific to CX3CR1 (Invitrogen, 702321), F4/80 (Cell Signaling Technology, 30325S), Ki67 (Cell Signaling Technology, 9129S), CC3 (Cell Signaling Technology, 9661S), SOX2 (Abcam, ab97959), GPNMB (Proteintech, 66926-1-Ig), Ly6C (BioLegend, 128024), and Ly6G (BioLegend, 127606) were used.

### Immunoblotting.

Protein expression in cells was assessed through immunoblotting analysis according to our previously established protocol (14, 17). Briefly, cells were lysed on ice using RIPA lysis buffer (Thermo Scientific, 89900) supplemented with Protease Inhibitor Cocktail (Thermo Scientific, 78429) and Phosphatase Inhibitor Cocktail (Cell Signaling Technology, 5870S). The protein concentration was determined using the BCA Protein Assay Kit (Thermo Fisher Scientific, PI23225). The protein solution was mixed with LDS sample buffer (GenScript, M00676) and heated at 95°C for 10 minutes. Subsequently, the protein samples were loaded onto SurePAGE gels (GenScript, M00653) and transferred to 0.2 μm nitrocellulose (NC) membranes (Bio-Rad, 1620112) using a predefined standard protocol for 30 minutes in the Trans-Blot Turbo system (Bio-Rad). The NC membranes were then blocked with 5% dry milk in TBST for 1 hour at room temperature and incubated overnight at 4°C with primary antibodies (diluted 1:1,000). After 3 washes, the membranes were incubated with HRP-conjugated anti-mouse (Cell Signaling Technology, 7076) or anti-rabbit (Cell Signaling Technology, 7074S) secondary antibodies for 1 hour at room temperature. Following another round of washing, the membranes were incubated with ECL substrate and imaged using the ChemiDoc Touch Imaging System (Bio-Rad). The antibodies used were sourced from specific companies and included β-actin (Cell Signaling Technology, 3700S), GPNMB (Proteintech, 66926-1-Ig), CD133 (Biosis, BS-4770R), SOX2 (Abcam, ab97959), P-RSK2 (Cell Signaling Technology, 3556S), RSK2 (Cell Signaling Technology, 9355S), P-PYK2 (Cell Signaling Technology, 3291S), PYK2 (Cell Signaling Technology, 3090S), and CD44 (Cell Signaling Technology, 37259S).

### Brain tumor immune cell isolation.

At the endpoint of experiments, tumor-bearing C57BL/6 mice were euthanized to retrieve their brains. Tumor-derived immune cells in the brain were isolated using the percoll density gradient cell separation method as we previously described (14, 17). In brief, after perfusion with PBS, brains were homogenized on ice with precold 10 mL HBSS (Gibco, 14025092). Then, cells were spun down at 500 g for 10 minutes at 4°C and were resuspended in 30% Percoll (GE Healthcare, 17-0891-01). The solution was gently laid on top of the 70% Percoll and centrifuged at 1,200*g* for 30 minutes at 4°C with accelerator 7 and breaker 0. After removing myelin and debris, the interphase was collected and centrifuged at 500 g for 10 minutes at 4°C. The tumor-derived cell pellet was resuspended for further analysis.

### Primary BMDMs and microglia isolation.

For mouse primary BMDM isolation, healthy C57BL/6 mice were euthanized by CO_2_ inhalation followed by cervical dislocation. The abdomen and hind legs of mice were sterilized with 70% ethanol before autopsy. An incision was made in the midline of the abdomen to expose the hind leg, and the skin and muscle of hind legs were removed as much as possible using scissors. The femur and tibia were isolated followed by 70% ethanol sterilization and 5 minutes of PBS washing. Ophthalmic scissors were used to carefully remove epiphyses of tibia and femur. Then, 5 mL RPMI containing 2% FBS and 1% antibiotic-antimycotic was injected from one end of the tibia and femur to flush out bone marrow cells. The collected cells were filtered through a 70 μm cell strainer and spun down at 500 g for 10 minutes at 4°C and were resuspended in RPMI containing 10% FBS, 1% antibiotic-antimycotic, and 1 ng/mL M-CSF for differentiation. Fresh media was added to the plate on day 3 to facilitate BMDM expansion. BMDMs were fully differentiated on day 6 and analyzed by flow cytometry analysis. For mouse primary microglia isolation, the percoll density gradient cell separation method (14, 17) was used, which was indicated above.

### Flow cytometry.

The single-cell suspensions were incubated with fixable viability dye (Invitrogen, 5211229035) on ice for 10 minutes. After washing with FACS buffer, cells were incubated with the TruStain FcX (anti-mouse CD16/32) Antibody (BioLegend, 103132) and True-Stain Monocyte Blocker (BioLegend, 426102) in 5% BSA PBST for 30 minutes on ice to block Fc receptors and nonspecific binding of the cyanine acceptor fluorophores. Different antibody cocktails, including Percp/Cy5.5 anti-mouse CD45 (BioLegend, 103132), PE/Cy7 anti-mouse/human CD11b (BioLegend, 101216), BV650 anti-mouse CD11c (BioLegend, 117339), Alexa Fluor 647 anti-mouse CD206 (BD Bioscience, 565250), PE anti-mouse CD68 (BD Bioscience, 566386), BV421 anti-mouse CX3CR1 (BD Bioscience, 567531), Alexa Fluor 700 anti-mouse Ly6C (BioLegend, 128024), and FITC anti-mouse Ly6G (BioLegend, 127606), were added to the samples and incubated for 30 minutes on ice. Intracellular protein staining was performed followed by cell surface marker staining. After washing with PBS, cells were incubated with fixation buffer (BioLegend, 420801) for 20 minutes. Cells were then permeabilized by permeabilization buffer (0.1% Triton X-100 in PBS). GPNMB primary antibody (Proteintech, 66926-1-Ig) was added to cell suspension for 1 hour on ice, followed by incubation with Alexa Fluor 594 anti-mouse secondary antibody (Cell Signaling Technology, 8890S) for 30 minutes. Cells were again incubated with fixation buffer overnight. Samples were read through the BD FACSymphony or BD LSRFortessa flow cytometer and analyzed in FlowJo v10.8.1.

### Proliferation (CFSE) assay.

Cell proliferation was assessed using the CellTrace carboxyfluorescein succinimidyl ester (CFSE) Cell Proliferation Kit (Invitrogen, C34554). Briefly, 1 × 10^6^ cells were collected and incubated with CFSE working solution (1:1,000) for 20 minutes at 37°C. The staining was stopped by adding complete cell culture media. After washing, cells were continuously cultured for 3–5 days with GPNMB recombinant protein treatment (R&D Systems, 2550-AC) or macrophage/microglia-derived CM in the dark and used for flow cytometry analysis. The percentage of CFSE-positive peaks over the undivided peak (generation 0) was analyzed using FlowJo v10.8.1.

### Tumorsphere formation assay.

The tumorsphere formation assay was performed as previously described (17). Briefly, GSCs were treated with GPNMB recombinant protein in the presence or absence of PF-271 (Selleck Chemicals, S2672) or SL0101 (MedChemExpress, HY-15237) or macrophage/microglia-derived CM. Cells were seeded into a 96-well plate at 100 cells/well with NSC medium, and tumorsphere numbers in each well were imaged and quantified after 2 weeks.

### Incucyte live-cell assay.

Cells were seeded in a 96-well plate (Corning, 3599) for culturing overnight. Cells were then treated with GPNMB recombinant protein in the presence or absence of PF-271 or SL0101 before reading in the IncuCyte Zoom Live cell analysis system (Sartorius). The cell proliferating rate was calculated by using the following formula: (cell confluency at indicated time – cell confluency at 0 hour)/cell confluency at 0 hour. The cells were monitored in the IncuCyte system for 48 hours.

### Metabolic assays.

L-Lactate levels were measured using the Glycolysis Assay Kit (Sigma-Aldrich, MAK439) according to the manufacturer’s instructions. Briefly, cells were seeded into a 6-well plate and treated with GPNMB recombinant protein in the presence or absence of PF-271 or SL0101 or macrophage/microglia-derived CM for 24 hours. Then, cells were cultured for another 24 hours in growth factor–free or FBS-free culture medium, and the glycolytic activity (L-lactate level) of media was measured at 565 nm wavelength. On the other hand, for the glucose metabolism, SeahorseXFe96 analyzer (Agilent) was used to determine ECARs. Glucose metabolism of indicated control and/or treated/modified cells was measured using the Seahorse XF Cell Mito Stress Test Kit (Agilent Technologies, 103015-100) in a Seahorse XFe96 on Seahorse XFe96/XF Pro FluxPak plates (Agilent Technologies, 103792-100). Briefly, cells were cultured in 6-well plate treated with GPNMB recombinant protein in the presence or absence of PF-271 or SL0101 or macrophage/microglia-derived CM before being plated onto seahorse cell culture plates coated with Cell-Tak (Corning, 354240). Cells were analyzed under basal conditions, and in response to sequential injections of glucose (25 mM), oligomycin (2 μM), FCCP (0.5 μM), and rotenone, antimycin A, and 2-Deoxy-D-glucose (1 μM, 1 μM and 50 mM) were used. Data were collected using Agilent Seahorse Wave 2.6 software.

### Enzyme-linked immunoassay (ELISA).

The levels of GPNMB in human plasma or CM of GSC272 educated HMC3 or THP-1 cells were measured by ELISA using a commercial GPNMB kit (MyBioSource, MBS7607219) following the manufacturer’s instructions. OD value was measured by a Biotek Synergy 2 SL Microplate Reader.

### Human phospho-kinase array.

After starvation for 24 hours, GSC2 cells were treated with 100 ng/mL GPNMB protein for 1 hour in an incubator. Protein lysis of GPNMB-treated GSC2 and control cells was prepared separately according to the instructions for the Proteome Profiler Human Phospho-Kinase Array Kit (Bio-Techne, ARY003C). Briefly, 400 mg protein in 300 mL lysis buffer from each group was mixed with Array Buffer (Bio-Techne, ARY003C) and incubated with both membranes A and B overnight at 4°C. Membranes were then washed in wash buffer and incubated with Detection Antibody Cocktail A or B (Bio-Techne, ARY003C) for 2 hours at room temperature, respectively. After washing, membranes were placed in an 8-well Multi-dish (Bio-Techne, ARY003C) containing the diluted Streptavidin-HRP (Bio-Techne, ARY003C) for 30 minutes on a rocking platform shaker. Chemiluminescent Reagent Mix (Bio-Techne, ARY003C) was prepared and added to membranes. Signal was determined using the ChemiDoc Imaging System (Bio-Rad) and quantified using ImageJ (NIH).

### Statistics.

Statistical analyses were performed with 2-tailed Student’s *t* tests for comparisons between 2 groups and 1-way ANOVA tests for comparisons among more than 2 groups. For the Incucyte recorded proliferation assay, a 2-way ANOVA test was used to determine the statistical difference between each proliferation curve. Data are represented as mean ± SEM. Correlation analysis was conducted using Pearson’s test to determine the Pearson correlation coefficient (*R* value) and *P* value. The survival analysis for animal models and patients with GBM was determined by conducting the log-rank (Mantel-Cox) test or Gehan-Breslow-Wilcoxon test. All statistical analyses were performed using GraphPad Prism 10. *P* values of less than 0.05 were considered significant.

### Study approval.

All animal experiments were performed with the approval of the IACUCs at Cleveland Clinic and Northwestern University.

### Data availability.

The data used to support the findings of this study are available within this article and within the Supporting Data Values file. The human TCGA GBM data (TCGA-GBM, TCGA-LGG, Rembrandt, and Gravendeel datasets) are available at GlioVis: https://gliovis.bioinfo.cnio.es/ The scRNA-Seq data of tumors from patients with GBM are available in public repositories, including EGAS00001004871 and EGAS00001004422 from EGA and GSE131928, GSE148842, GSE89567 and GSE182109 from GEO.

## Author contributions

YL performed most experiments. YL and LP performed single-cell sequencing data analysis. LP performed CD44-knockdown experiments. FK performed Seahorse experiments. JW and FZ helped with immunoblotting experiments. CH provided samples from patients with GBM and diagnosed the patients according to the WHO diagnostic criteria. SB, JSY, and JDL provided feedback and comments on this study. PC designed the project. YL and PC wrote the manuscript. All authors participated in editing the manuscript.

## Supplementary Material

Supplemental data

Unedited blot and gel images

Supplemental table 1

Supporting data values

## Version 1 07/08/2025

Electronic publication
