# Supplementary material for: Glycoprotein NMB mediates bidirectional GSC-TAM interactions to promote tumor progression
Source: JCI Insight. 2025 Jul 8;10(13):e187684. doi: 10.1172/jci.insight.187684 (PMC12288892; doi:10.1172/jci.insight.187684)
Supplement: Supplemental data [file jciinsight-10-187684-s147.pdf]

Supplemental Figure 1

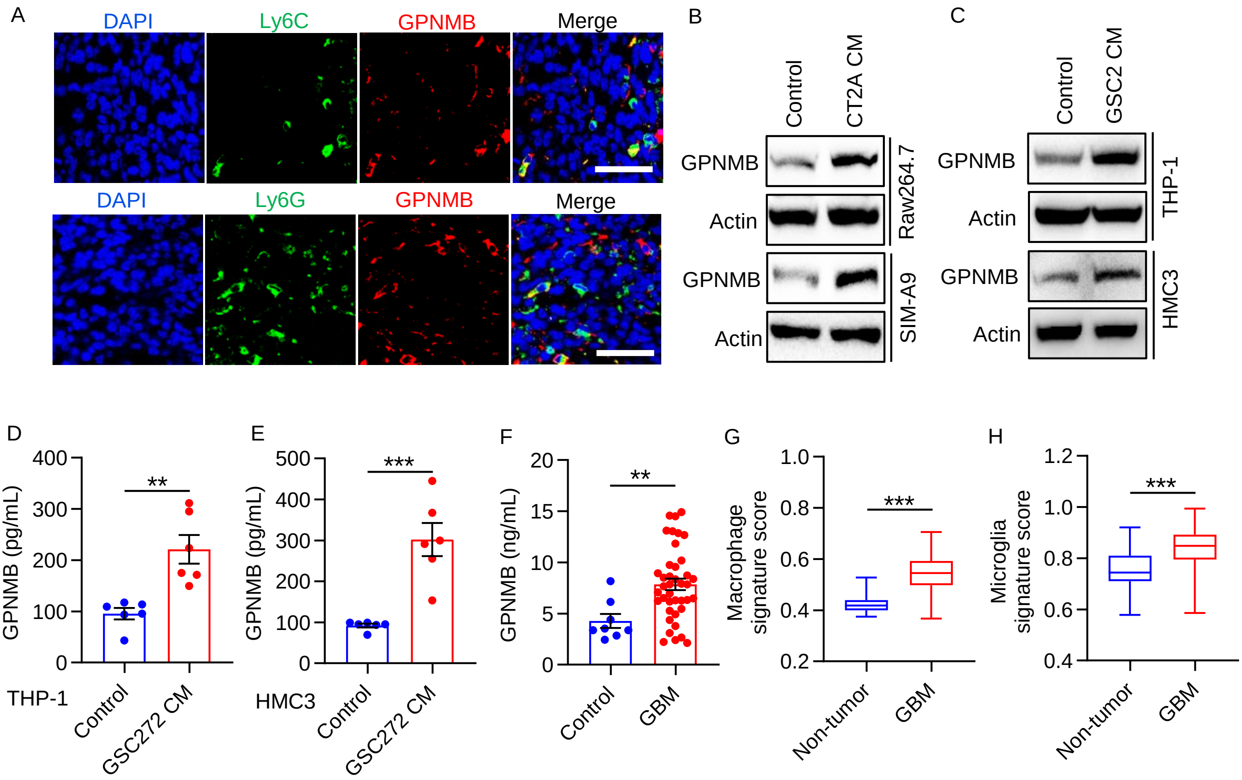

Supplemental Figure 1. GPNMB is highly expressed in TAMs of GBM

(A) Co-immunofluorescence staining for GPNMB (red) and Ly6C (monocytic immature myeloid cell marker, green) or Ly6G (polymorphonuclear immature myeloid cell marker, green) in CT2A tumors implanted in C57BL/6 mice. Scale bar, 50 μm. **(B)** Immunoblots for GPNMB in lysates of Raw264.7 macrophages and SIM-A9 microglia treated with the conditioned media (CM) of CT2A cells for 24 hours. **(C)** Immunoblots for GPNMB in lysates of THP-1 macrophages and HMC3 microglia treated with GSC2 CM for 24 hours. **(D and E)** ELISA for GPNMB in the CM of THP-1 macrophages (D) and HMC3 microglia (E) treated with GSC272 CM for 24 hours. n = 6 independent samples. Student's t test. **(F)** ELISA for GPNMB in the plasma from healthy controls (n = 8) and patients with GBM (n = 40). Student's t test. **(G and H)** Macrophage (G) or microglia (H) signature scores in healthy controls (n = 28) and patients with GBM (n = 219) from Rembrandt dataset. Student's t test. **, $P < 0.01$, ***, $P < 0.001$.

Supplemental Figure 2

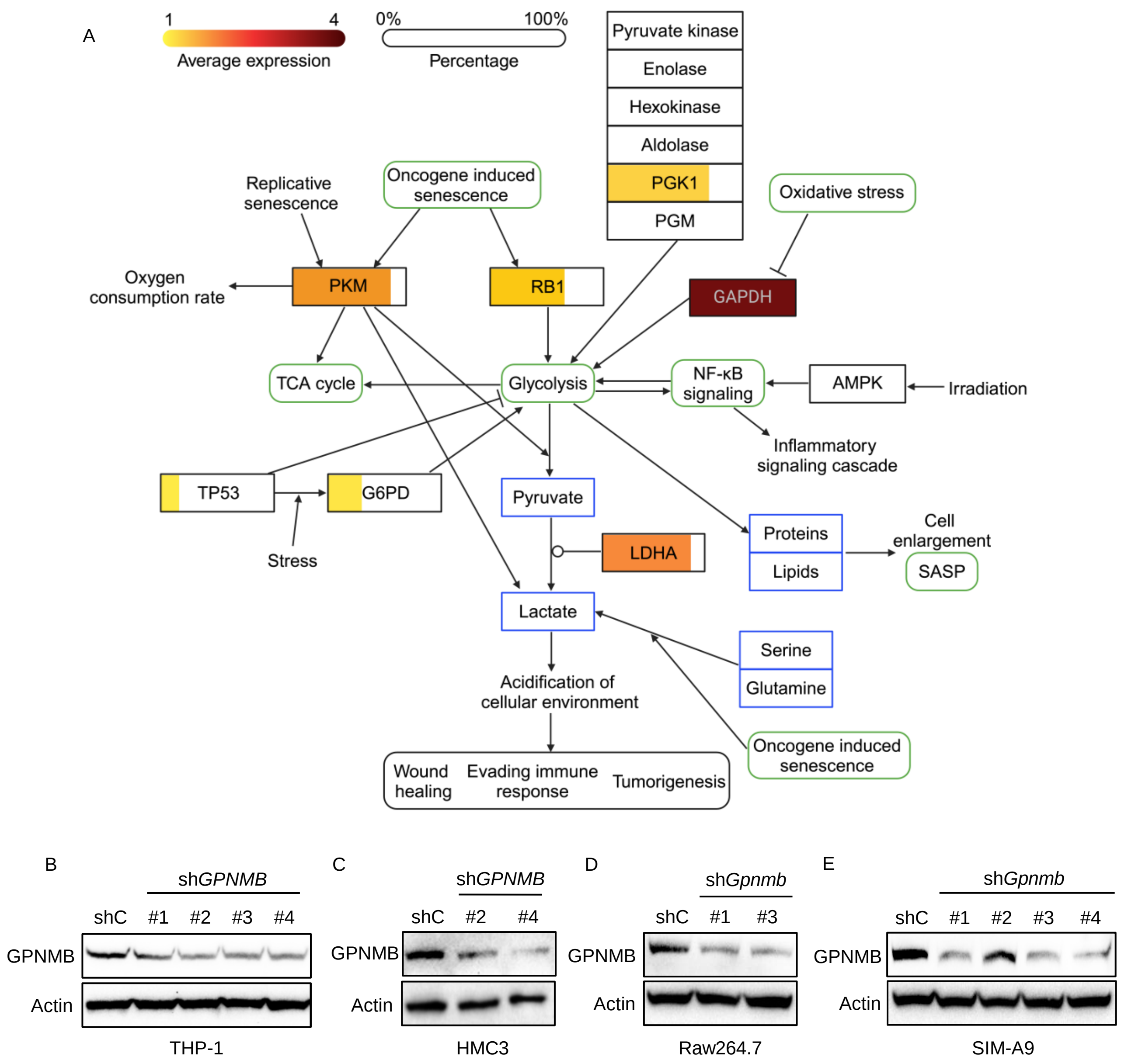

Supplemental Figure 2. The glycolysis of GSCs is facilitated by GPNMB

(A) Correlated pathway interaction network of glycolysis with the expression levels of key genes in GBM tumors with *GPNMB*-high compared with *GPNMB*-low in macrophages based on single-cell RNA sequencing dataset (EGAS00001004871). **(B and C)** Immunoblots for GPNMB in lysates of THP-1 macrophages (B) and HMC3 microglia (C) expressing shRNA control (shC) or *GPNMB* shRNA (sh*GPNMB*). **(D and E)** Immunoblots for GPNMB in lysates of Raw264.7 macrophages (D) and SIM-A9 microglia (E) expressing shC and sh*Gpnmb*.

Supplemental Figure 3

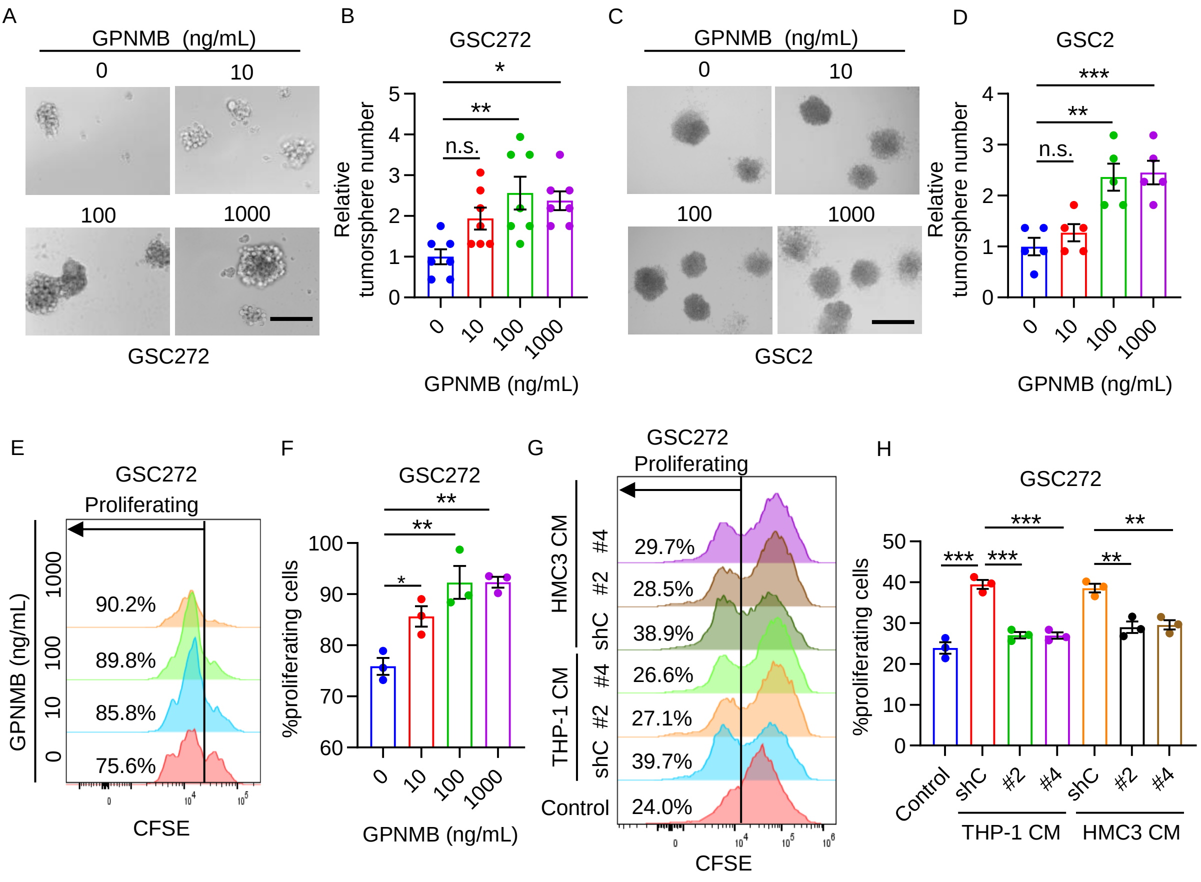

Supplemental Figure 3. GPNMB increased the GSC self-renewal

(A and B) Representative images (A) and quantification (B) of tumorspheres of GSC272 treated by GPNMB recombinant protein in different indicated concentrations for 2 weeks. Scale bar, 200 μm. n = 7 independent samples. One-way ANOVA test. **(C and D)** Representative images (C) and quantification (D) of tumorspheres of GSC2 treated by GPNMB recombinant protein in different indicated concentrations for 2 weeks. Scale bar, 200 μm. n = 5 independent samples. One-way ANOVA test. **(E and F)** Representative images (E) and quantification (F) of proliferation of GSC272 treated by GPNMB recombinant protein in different indicated concentration for 5 days. n = 3 independent samples. One-way ANOVA test. **(G and H)** Representative images (G) and quantification (H) of proliferation of GSC272 treated by the conditioned media (CM) of THP-1 macrophages or HMC3 microglia expressing shRNA control (shC) or *GPNMB* shRNA (sh*GPNMB*) for 3 days. n = 3 independent samples. One-way ANOVA test. *, $P < 0.05$, **, $P < 0.01$, ***, $P < 0.001$, n.s., not significant ($P > 0.05$).

Supplemental Figure 4

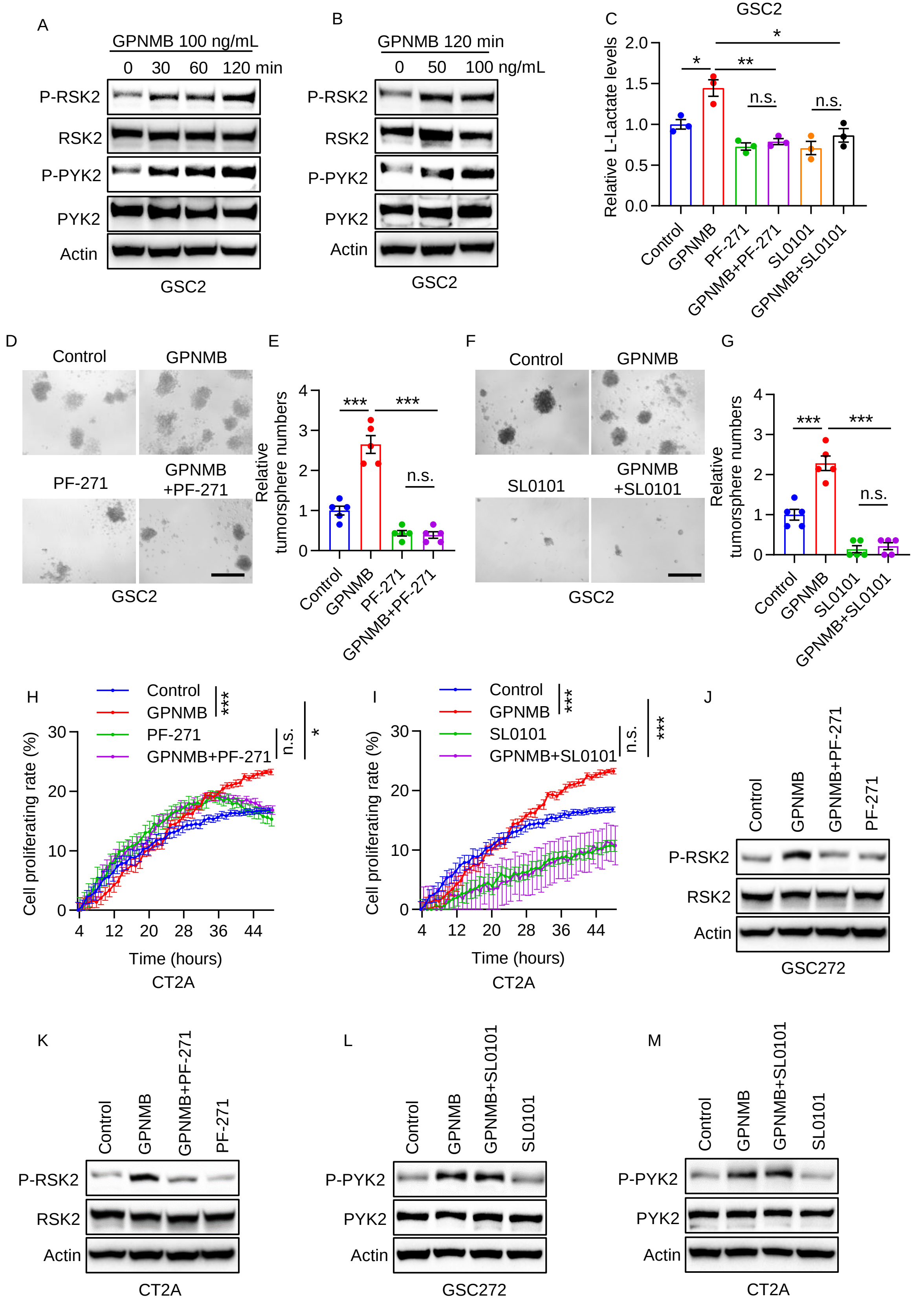

Supplemental Figure 4. GPNMB activates PYK2-RSK2 axis to enhance GSC stemness

(A and **B)** Immunoblots for P-RSK2, RSK2, P-PYK2, and PYK2 in lysates of GSC2 treated by GPNMB recombinant protein at indicated concentrations and timepoints. **(C)** Quantification of relative L-Lactate levels in GSC2 treated by GPNMB recombinant protein (100 ng/mL) in the presence or absence of PYK2 inhibitor PF-271 (15 nM) or RSK1/2 inhibitor SL0101 (100 μM) for 24 hours. n = 3 independent samples. One-way ANOVA test. **(D** and **E)** Representative images (D) and quantification (E) of tumorspheres of GSC2 treated by GPNMB recombinant protein (100 ng/mL) in the presence or absence of PF-271 (15 nM) for 2 weeks. Scale bar, 200 μm. n = 5 independent samples. One-way ANOVA test. **(F** and **G)** Representative images (F) and quantification (G) of tumorspheres of GSC2 treated by GPNMB recombinant protein (100 ng/mL) in the presence or absence of SL0101 (100 μM) for 2 weeks. Scale bar, 200 μm. n = 5 independent samples. One-way ANOVA test. **(H** and **I)** Proliferation of CT2A cells treated by GPNMB recombinant protein (100 ng/mL) in the presence or absence of PF-271 (15 nM) (H) and SL0101 (100 μM) (I) at indicated timepoints. n = 8 independent samples. Two-way ANOVA test. **(J** and **K)** Immunoblots for P-RSK2 and RSK2 in lysates of GSC272 (J) and CT2A cells (K) treated by GPNMB recombinant protein (100 ng/mL) in the presence or absence of PF-271 (15 nM) for 2 hours. **(L** and **M)** Immunoblots for P-PYK2 and PYK2 in lysates of GSC272 (L) and CT2A cells (M) treated by GPNMB recombinant protein (100 ng/mL) in the presence or absence of SL0101 (100 μM) for 2 hours. *, $P < 0.05$, **, $P < 0.01$, ***, $P < 0.001$, n.s., not significant ($P > 0.05$).

Supplemental Figure 5

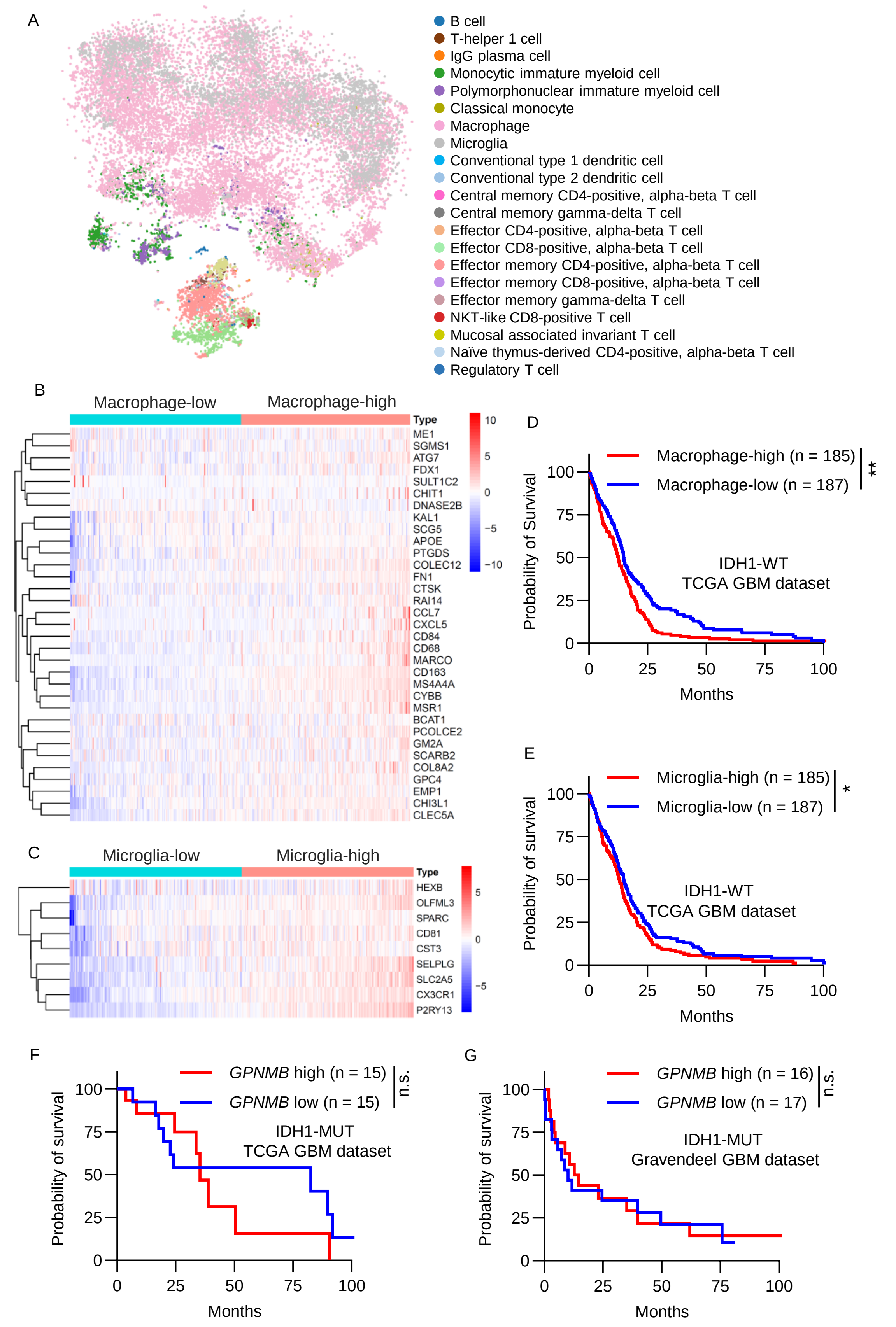

Supplemental Figure 5. The relationship between TAMs and GBM patient survival

(A) T-distributed stochastic neighbor embedding (t-SNE) dimensional reduction of immune cells from GBM tumors based on single-cell RNA sequencing (scRNA-seq) dataset (EGAS00001004871). **(B)** Clustering of IDH1-WT human TCGA GBM samples into macrophage-high and macrophage-low groups using macrophage gene signature. **(C)** Clustering of IDH1-WT human TCGA GBM samples into microglia-high and microglia-low groups using microglia gene signature. **(D** and **E)** Survival curves of IDH1-WT TCGA GBM patients with high and low macrophage (D) and microglia (E) signatures. Log-rank test. **(F** and **G)** Survival curves of GBM patients with high and low *GPNMB* expression in IDH1-mutant TCGA (F) and Gravendeel (G) GBM datasets. Log-rank test. *, $P < 0.05$, **, $P < 0.01$. n.s., not significant ($P > 0.05$).
