# Supplementary material for: Glycoprotein NMB mediates bidirectional GSC-TAM interactions to promote tumor progression
Source: JCI Insight. 2025 Jul 8;10(13):e187684. doi: 10.1172/jci.insight.187684 (PMC12288892; doi:10.1172/jci.insight.187684)

Full unedited blot for Figure 1

Figure 1L

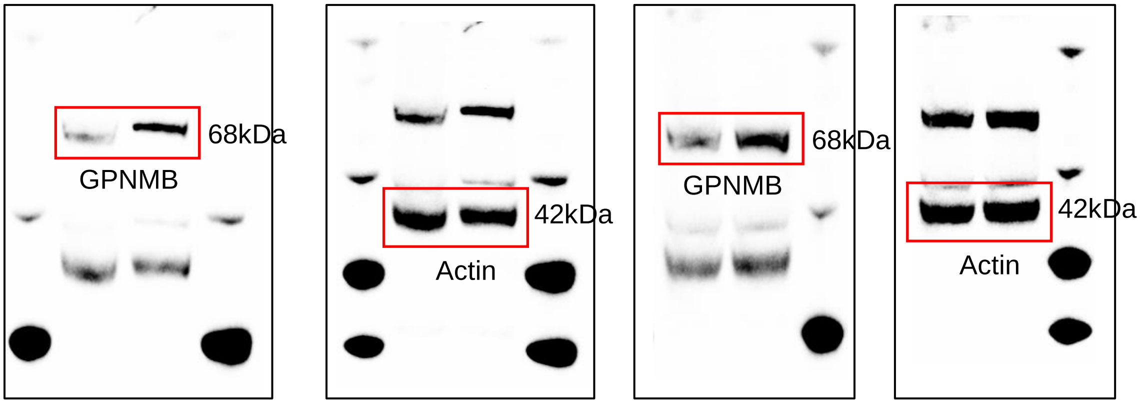

Figure 1M

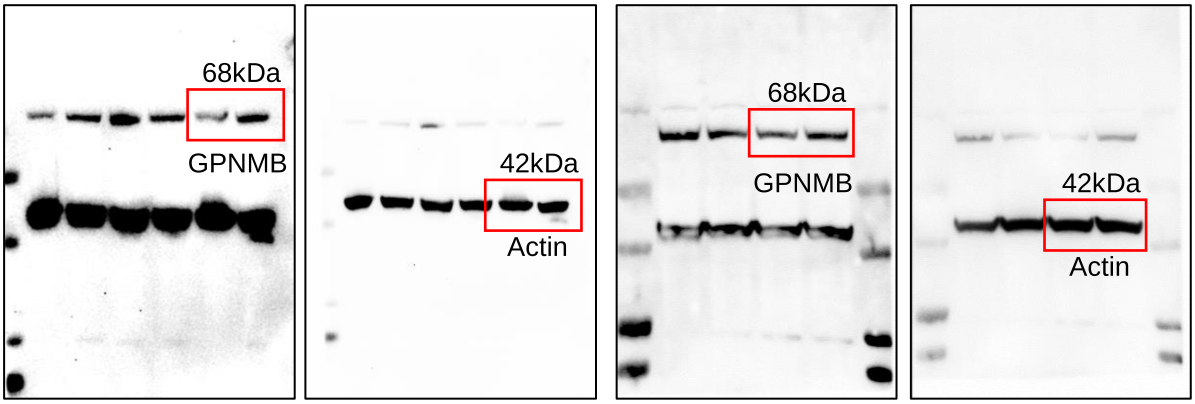

Full unedited blot for Figure S1

Figure S1B

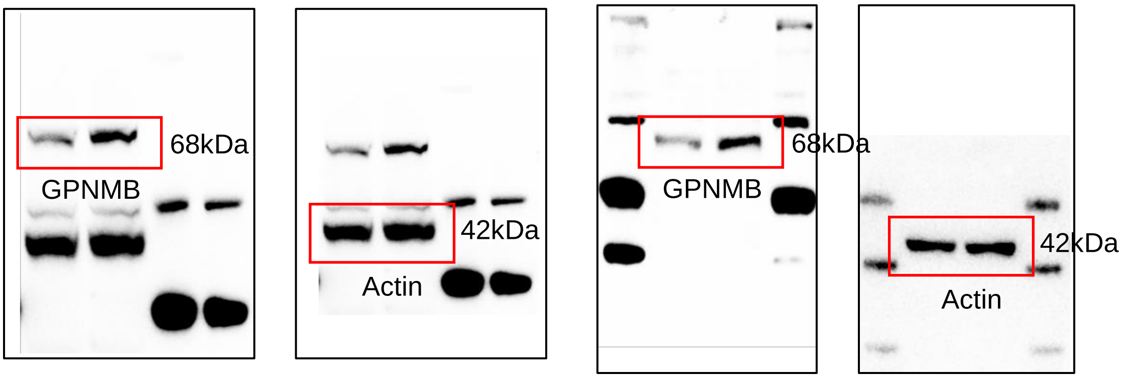

Figure S1C

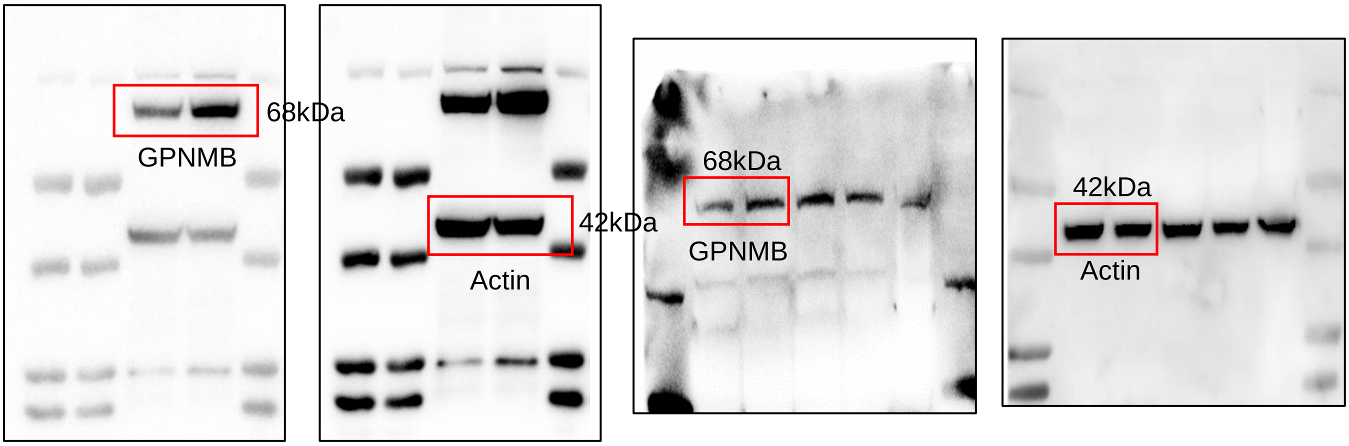

Full unedited blot for Figure S2

Figure S1B

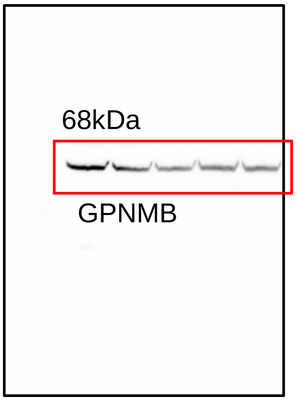

Figure S2C

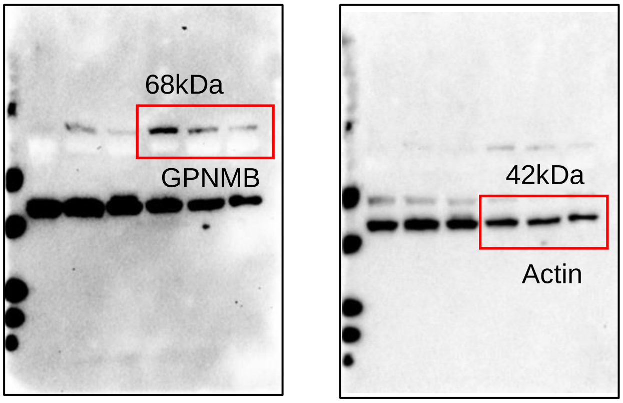

Figure S1D

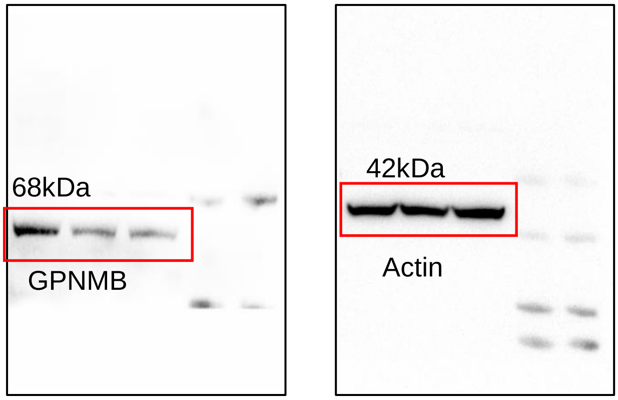

Figure S2E

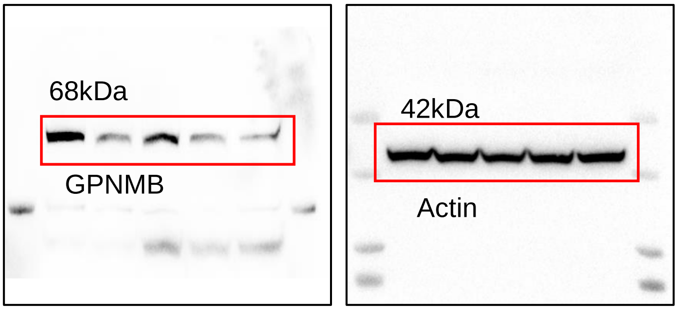

Full unedited blot for Figure 3

Figure 3C

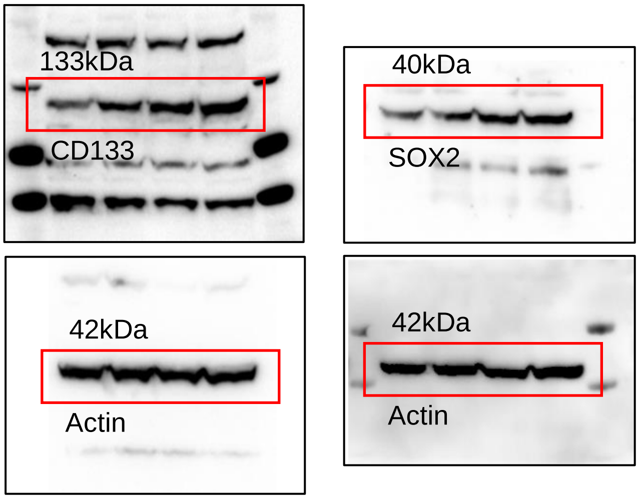

Figure 3D

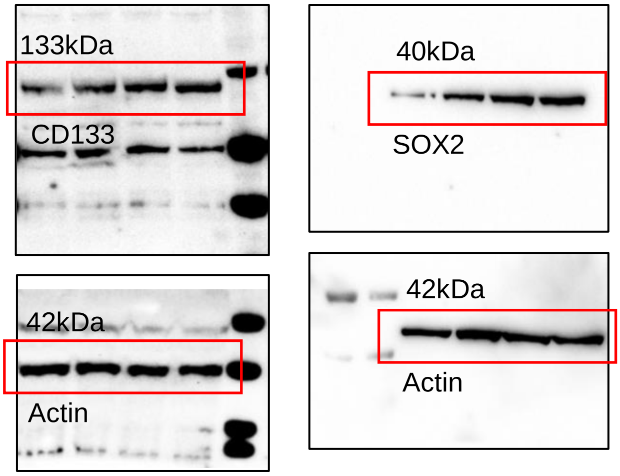

Figure 3E

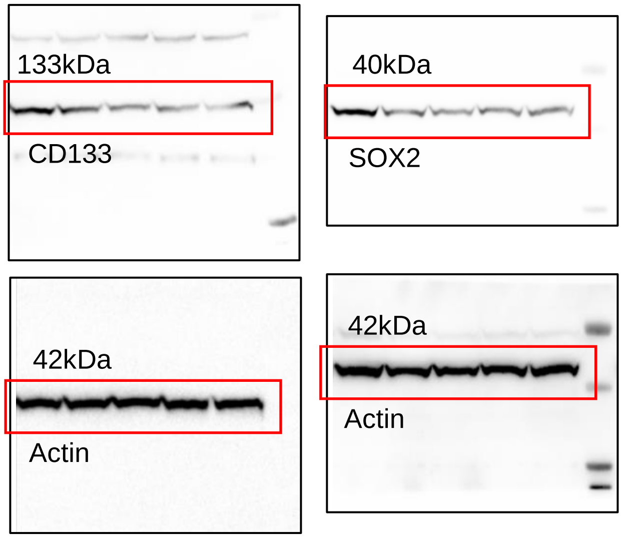

Figure 3F

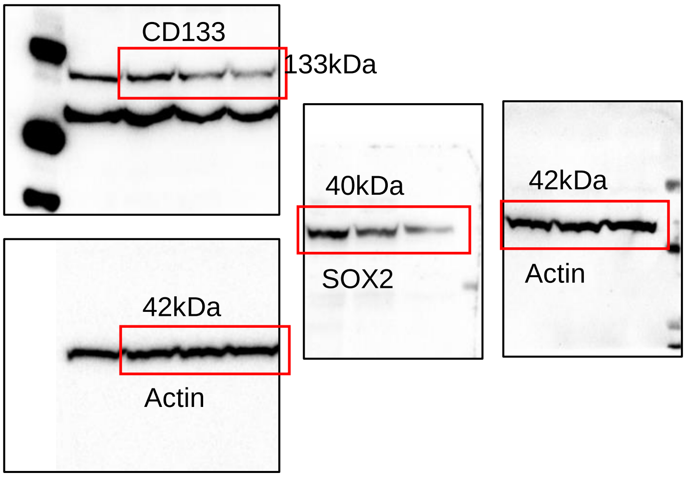

Figure 3G

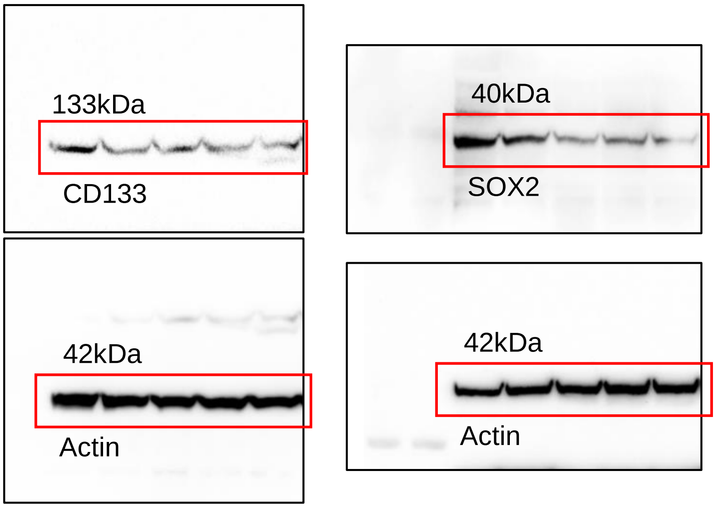

Figure 3H

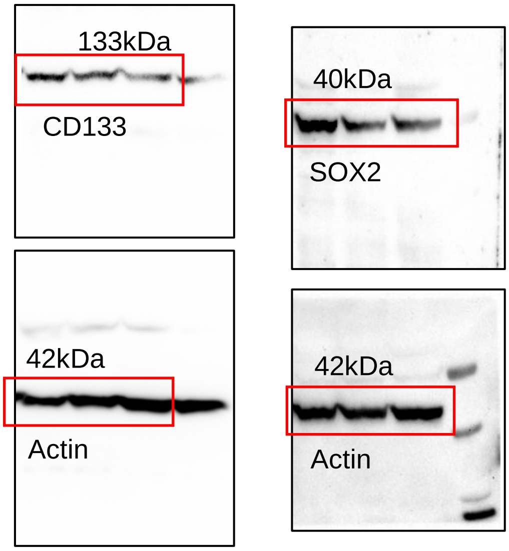

Full unedited blot for Figure 4

Figure 4C

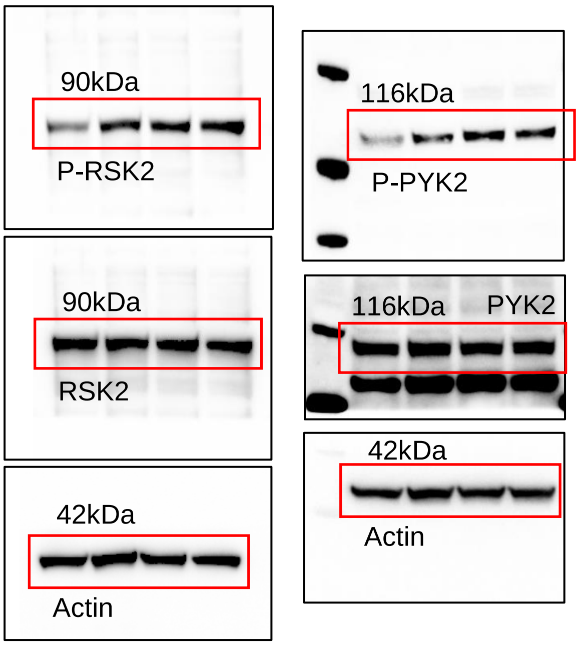

Figure 4D

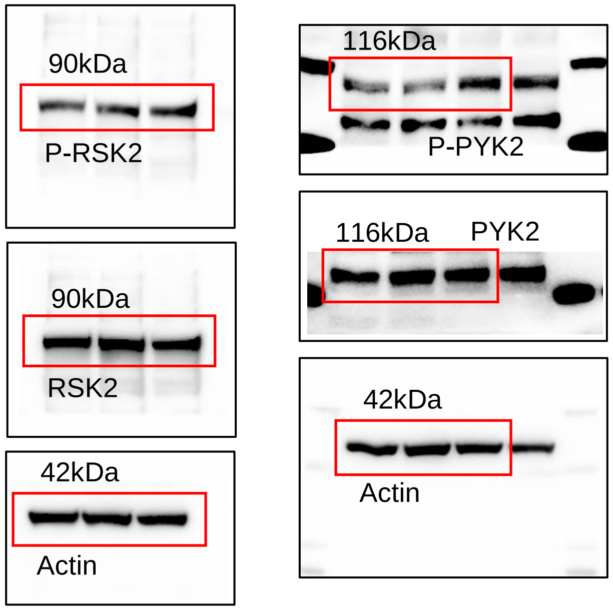

Figure 4I

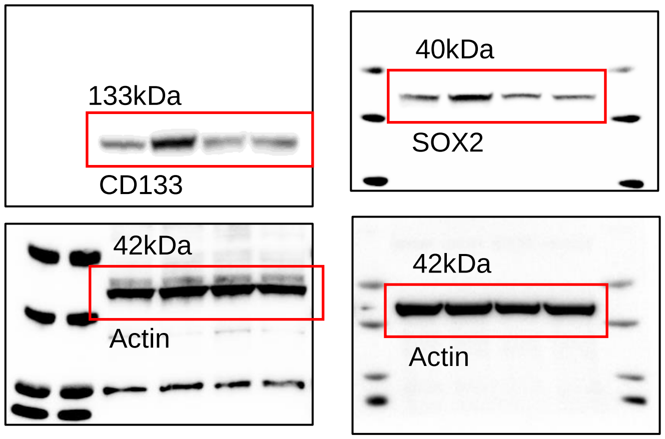

Figure 4J

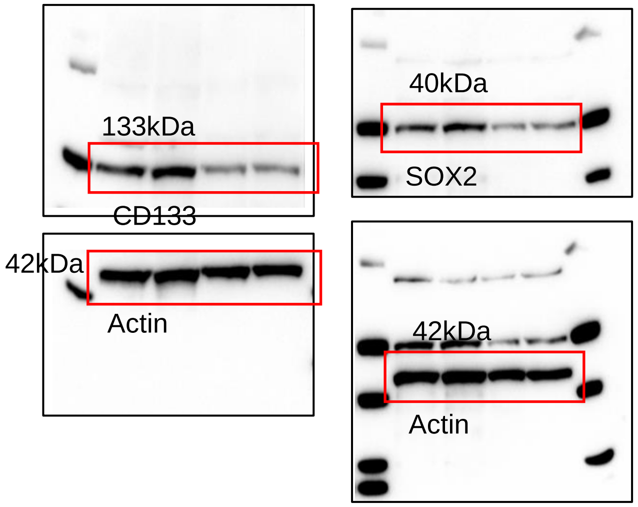

Full unedited blot for Figure S4

Figure S4A

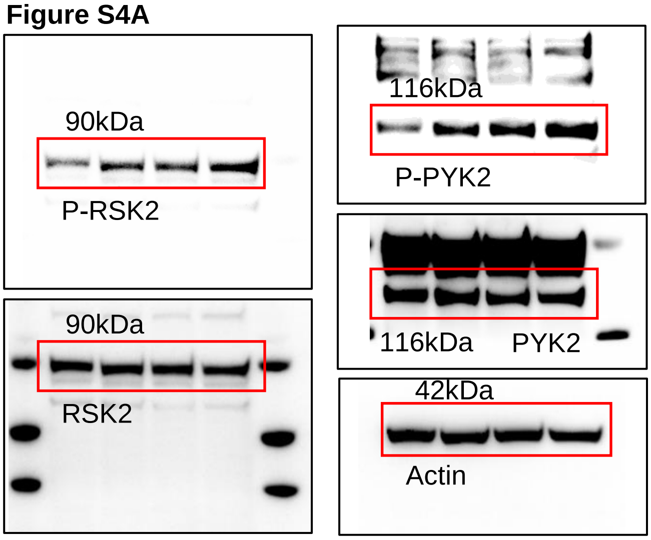

Figure S4B

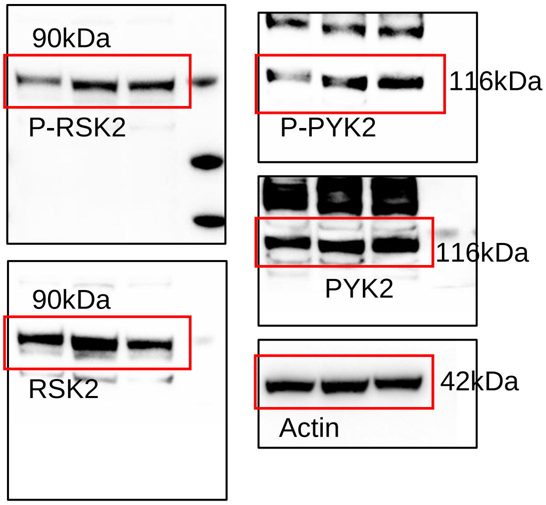

Figure S4J

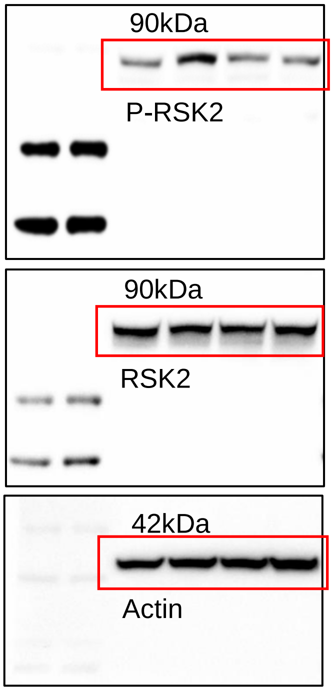

Figure S4K

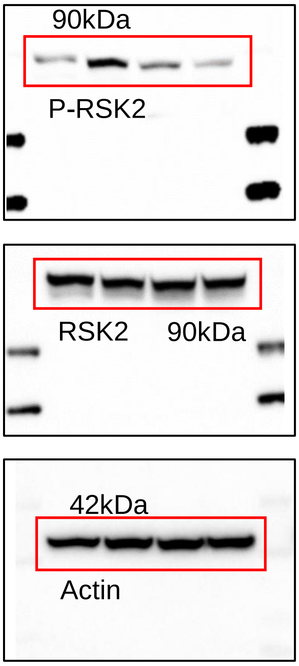

Figure S4L

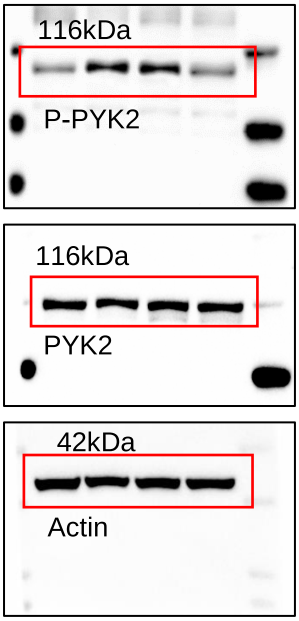

Figure S4M

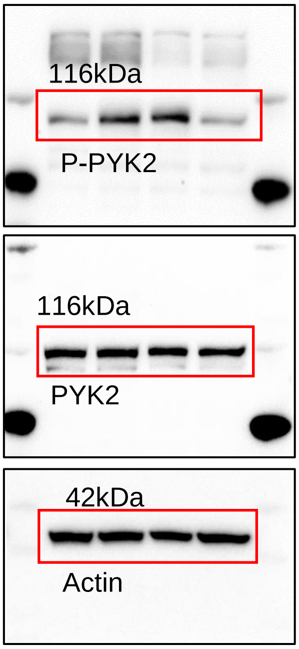

Full unedited blot for Figure 5

Figure 5E

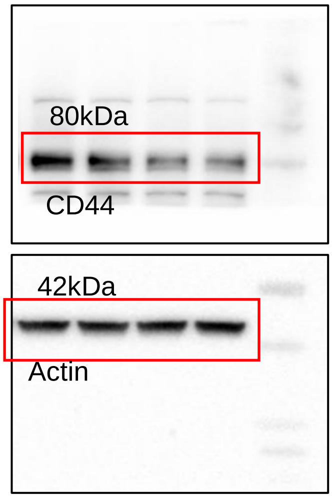

Figure 5F

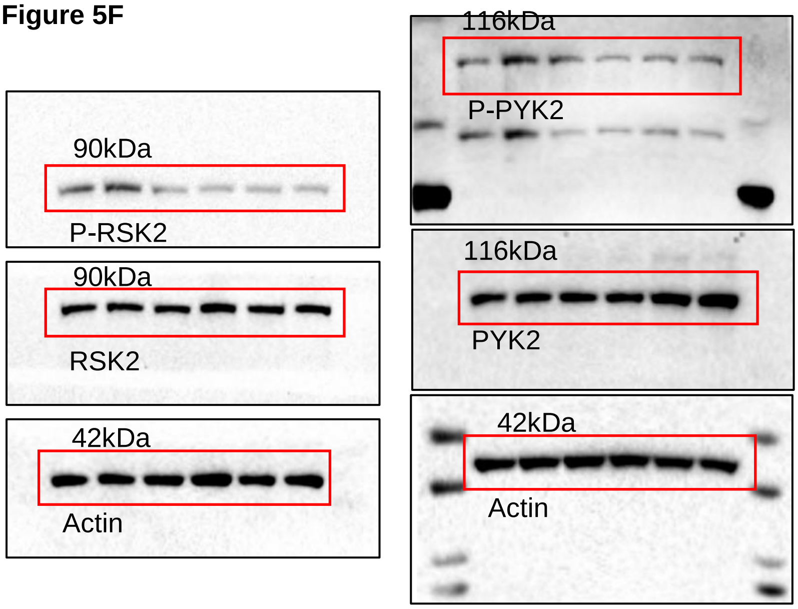

Figure 5H

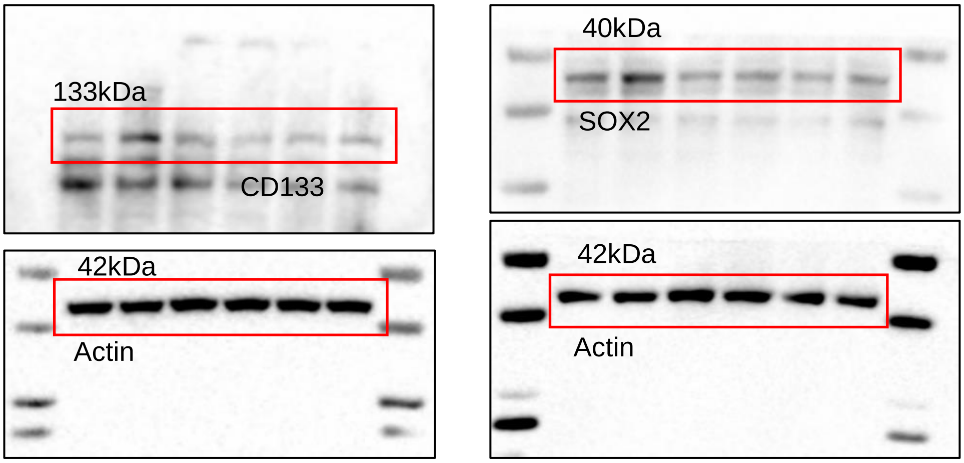

Supplement: Unedited blot and gel images [file jciinsight-10-187684-s148.pdf]
